# Life‐history strategy and extinction risk in the warm desert perennial spring ephemeral *Astragalus holmgreniorum* (Fabaceae)

**DOI:** 10.1002/ece3.8301

**Published:** 2021-11-05

**Authors:** Renée Van Buren, Allyson B. Searle, Susan E. Meyer

**Affiliations:** ^1^ Department of Biology Utah Valley University Orem Utah USA; ^2^ Shrub Sciences Laboratory USDA Forest Service Rocky Mountain Research Station Provo Utah USA

**Keywords:** endangered species, fast‐slow continuum, hemicryptophyte, matrix modeling, Mojave Desert, persistent seed bank, population viability analysis, spring ephemeral

## Abstract

This study of *Astragalus holmgreniorum* examines its adaptations to the warm desert environment and whether these adaptations will enable it to persist. Its spring ephemeral hemicryptophyte life‐history strategy is unusual in warm deserts. We used data from a 22‐year demographic study supplemented with reproductive output, seed bank, and germinant survival studies to examine the population dynamics of this species using discrete‐time stochastic matrix modeling. The model showed that *A*. *holmgreniorum* is likely to persist in the warm desert in spite of high dormant‐season mortality. It relies on a stochastically varying environment with high inter‐annual variation in precipitation for persistence, but without a long‐lived seed bank, environmental stochasticity confers no advantage. Episodic high reproductive output and frequent seedling recruitment along with a persistent seed bank are adaptations that facilitate its survival. These adaptations place its life‐history strategy further along the spectrum from “slower” to “faster” relative to other perennial spring ephemerals. The extinction risk for small populations is relatively high even though mean *λ*_s_ > 1 because of the high variance in year quality. This risk is also strongly dependent on seed bank starting values, creating a moving window of extinction risk that varies with population size through time. *Astragalus holmgreniorum* life‐history strategy combines the perennial spring ephemeral life form with features more characteristic of desert annuals. These adaptations permit persistence in the warm desert environment. A promising conclusion is that new populations of this endangered species can likely be established through direct seeding.

## Graphical abstract

We used a 22‐year demographic dataset to produce a matrix population model for *Astragalus holmgreniorum*, an endangered Mojave Desert perennial with an unusual life‐history strategy. We demonstrated that the species depends on a stochastic environment for persistence. Favorable years permit high seed production and high recruitment success, while unfavorable years are tolerated through the existence of a long‐lived seed bank. This perennial spring ephemeral is short‐lived and has many life‐history features in common with desert annuals, increasing its probability of survival in the warm desert environment.

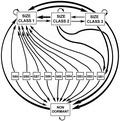

## 1 INTRODUCTION

A major goal of plant evolutionary ecology is to understand patterns of life‐history evolution (Gadgil & Solbrig, 1972; Grime, 1977; Pianka, 1970). Life‐history theory is usually explained using examples from species with life histories that represent strong contrasts, but many species have life histories intermediate among these extremes. How selection might operate to shift a species with an intermediate life‐history strategy in one direction or another along these continua is less commonly considered.

Population matrix modeling is a valuable approach for integrating demographic data into an analytical framework in order to understand plant life histories (Caswell, 2001; Morris & Doak, 2002). These models use multiple years of demographic data from the field to calculate transition probabilities for vital rates important in the life history of a species. These are combined into matrix elements that represent transitions among the stage or age classes in the life cycle diagram. The traditional approach to modeling plant life history has used deterministic models that include only mean values for vital rates and thus for elements of the transition matrix. A deterministic model converges to a single asymptotic value, the deterministic population growth rate (*λ*_d_). These models can be useful as heuristic tools, as well as for plant species where environmental drivers and consequently vital rates do not vary widely and where the mean condition permits persistence (Crone et al., 2011). For plant species that grow in stressful environments with high inter‐annual variation in year quality, however, deterministic models are not good predictors of population growth.

Stochastic population matrix models incorporate vital rate variances and covariances as well as means into the estimate of population growth rate and are thus better able to predict the consequences of environmental variation on population growth (Morris & Doak, 2002). The result of each iteration of a stochastic model is an estimate of the stochastic population growth rate (*λ*_s_). It is based on the random draw of values from a set of vital rate probability distributions to populate the matrix for each time step (Morris & Doak, 2002).

A considerable body of theoretical and empirical evidence has supported the idea that mean *λ*_s_ will almost always be lower than *λ*_d_, that is, that adding vital rate variation to the model will slow population growth (Lewontin & Cohen, 1969; Tuljapurkar & Orzack, 1980). This is because population growth as specified in these models is a multiplicative process that is more sensitive to bad years than good years. More recent work has shown that this constraint may be relaxed if certain assumptions are not met. These include the assumptions that vital rates and log‐*λ*_s_ are linearly related and that the mean condition permits population persistence, that is, *λ*_d_ ≥ 1 (Boyce et al., 2006; Drake, 2005; Morris & Doak, 2004).

More recently, the review by Lawson et al. (2015) examined both theoretical and empirical evidence to investigate the conditions under which increased environmental variance (stochasticity) could increase the population growth rate. They found that the effect of environmental stochasticity on population growth rate is determined by the shape of the relationships between population growth rate and independent variables that are related to environmental quality. When this relationship is concave, increased stochasticity decreases the mean population growth rate as predicted by classical models (Lawson et al., 2015, Figure 2). When the relationship is linear, environmental stochasticity has no effect. However, when the relationship between population growth rate and environmental quality is convex, stochasticity can increase the population growth rate. Lawson et al. (2015) also discuss the idea that different vital rates could have response curves with different relationships to environmental quality, and that these could interact to increase the positive effect of stochasticity on population growth rate. “Labile” vital rates, (e.g., reproductive output) could potentially increase nonlinearly with environmental quality, accelerating population growth in years when environmental quality is higher. These could be combined with “buffering” vital rates (e.g., seed dormancy loss rate) that are insensitive to changes in environmental quality and thus provide protection against population decline in unfavorable years.

**FIGURE 1:**
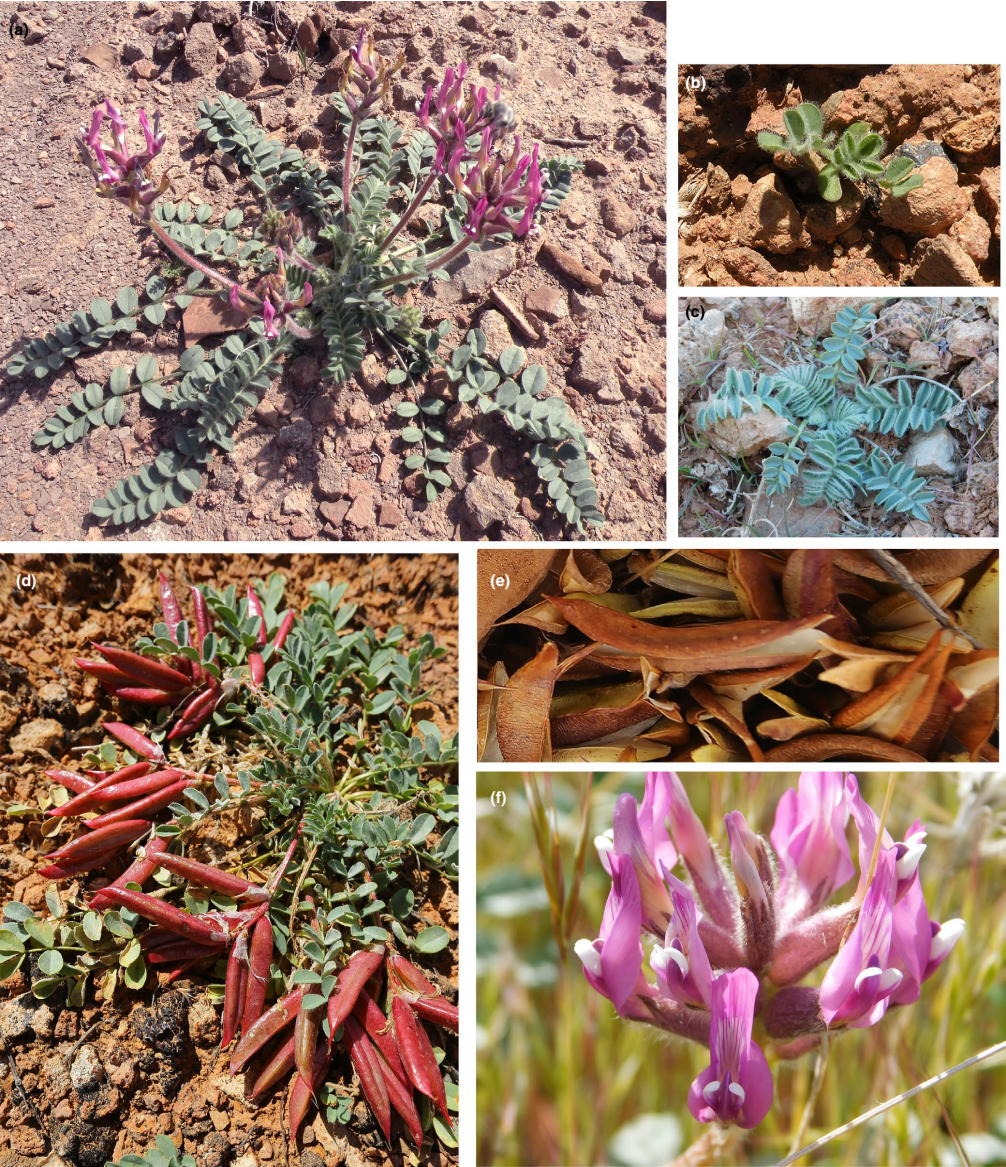
*Astragalus holmgreniorum* life history: (a) Flowering adult, (b) Recruited seedling, (c) Returning adult, (d) Adult in fruit, (e) Mature fruits beginning to dehisce, and (f) Close‐up of flowers. (Photo credits: a: Susan Meyer, b, c: Alyson DeNittis, d, e: Jonathan Barth, f: Sydney Houghton)

In this study, we examine the life history of the warm desert endemic *Astragalus holmgreniorum* Barneby (Figure 1) to ask how well the life history of this species permits it to persist in its warm desert environment and whether it has evolved specific traits that comprise a warm desert‐adapted life‐history strategy. Warm deserts are stressful environments with high inter‐annual variation in quality that is mediated by large and unpredictable variation in precipitation around a generally low mean.

*Astragalus holmgreniorum* has a hemicryptophyte spring ephemeral life history (Rominger et al., 2019) that is unusual in the warm desert. Warm desert floras worldwide show a bimodal life‐history strategy distribution dominated by stress‐tolerant shrubs and annual plants (Danin & Orshan, 1990; Pierce et al., 2017; Whittaker & Niering, 1964). Hemicryptophyte spring ephemerals, that is, spring ephemeral herbaceous perennial species that position their dormant meristems at or very near the soil surface, are especially uncommon. The soil surface in warm deserts can be many degrees hotter in summer than either the air above the surface or the soil at a depth of a few centimeters (Geiger et al., 2009), adding another element of stress and posing a significant risk of low dormant‐season survival for these species.

As mentioned above, divergent life‐history strategies have evolved in response to the constraints of the warm desert environment. Desert shrubs have evolved to tolerate the stressful summer season by raising their meristems well above the hot soil surface, as well as through numerous other morphological and physiological mechanisms for reducing or tolerating both heat and water stress (Peguero‐Pina et al., 2020). They represent the stress‐tolerator corner on the Grime life‐history triangle (Grime, 1977). This is an essentially K‐selected life‐history strategy (Gadgil & Solbrig, 1972) that emphasizes a long life span over recruitment through sexual reproduction. Seed production may only occur sporadically, and persistent seed banks are rare.

In contrast, the desert annual life‐history strategy is closer to the ruderal corner of the Grime life‐history scheme (Grime, 1977). These species are similar to R‐selected ruderal species (Gadgil & Solbrig, 1972) in that they establish and produce seeds quickly in favorable environments that are only available for short periods. Ruderal species may find new favorable environments in space, through dispersal, or in time, through persistent seed banks. Warm desert annuals are also able to complete their life cycles quickly during the most favorable season and thereby escape the stresses of the unfavorable season as seeds. Because years show extreme variation in quality in the warm desert, this life‐history strategy includes the ability to maximize both recruitment and seed production in favorable years and to survive multiple years of unfavorable conditions in the persistent seed bank (Pake & Venable, 1996).

Perennial spring ephemerals in warm deserts are similar to annuals in that they grow actively only in the most favorable season and escape the stressful season through dormancy. Most of these are long‐lived geophytes with deeply buried dormant meristems that are not exposed to the extreme heat of the surface soil. As in desert shrubs, the emphasis for most desert geophytes is on longevity and sometimes clonal reproduction, not sexual reproduction (Fragman & Shmida, 1997). We considered whether *A*. *holmgreniorum* would compensate for its increased dormant‐season mortality risk by sharing more life‐history traits with desert annuals, that is, episodic high seed production, successful recruitment in favorable years, and a long‐lived seed bank, rather than exhibiting life‐history traits shared with more typical desert perennial plants.

**FIGURE 2:**
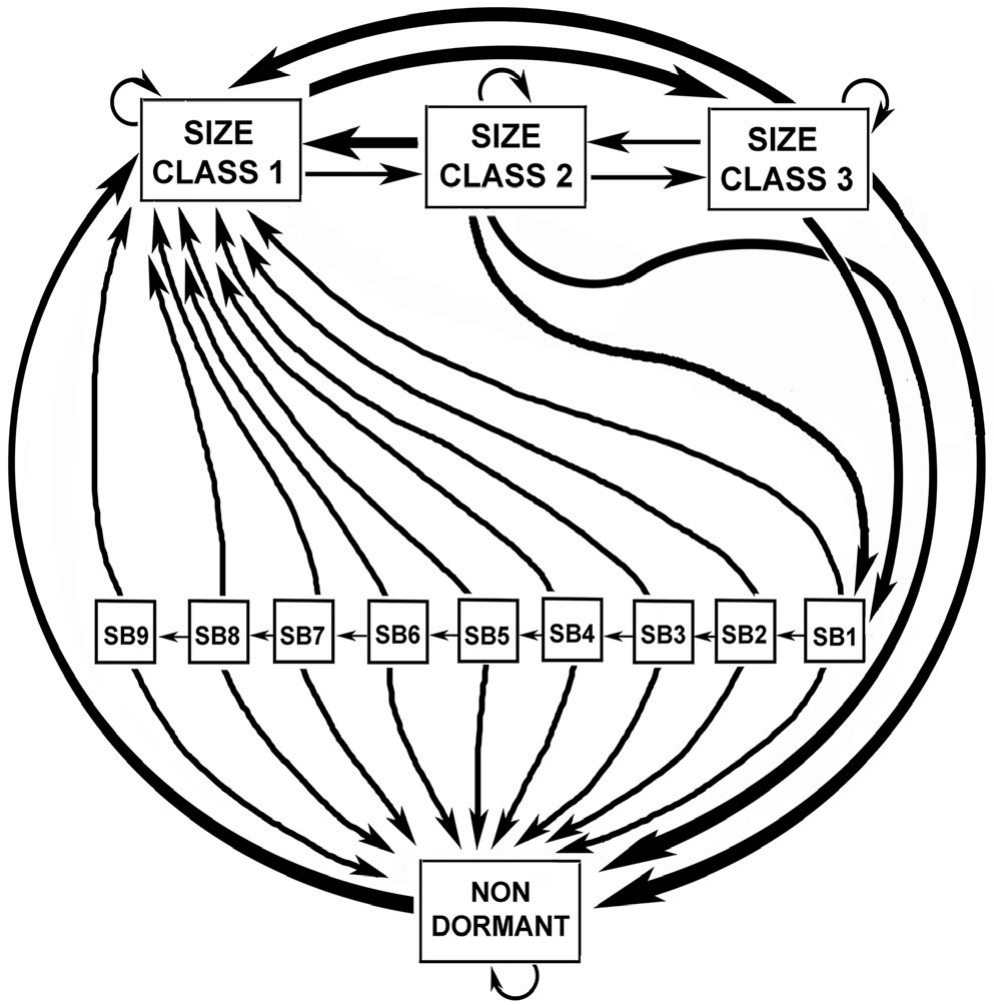
Life cycle diagram for *Astragalus holmgreniorum*. Square boxes represent life stages between which plants can transition each year. These include three size classes (S1–S3), dormant seeds of different ages in the persistent seed bank (SB1–SB9), and nondormant carryover seeds (NONDORMANT). See Table 1 for vital rates contributing to each transition

We used data from a 22‐year demographic study supplemented with information on reproductive output and seed bank dynamics to examine population fluctuations through time and to construct a discrete‐time stochastic population matrix model utilizing a life cycle diagram for *A*. *holmgreniorum* based on our field data for this species (Figure 2). We then used the resulting model along with the demographic dataset to address five principal hypotheses:

1. Vital rates underlying large population fluctuations through time are correlated with stochastic variation in yearly environmental quality that can be quantified as seasonal precipitation variation. Vital rates are therefore expected to show the same wide variance that is evident for precipitation in this highly stochastic desert environment.
2. Environmental conditions in an average year in the warm desert habitat are below the threshold for the survival of this species (i.e., *λ*_d_ < 1), so that increased environmental stochasticity (higher inter‐annual variance) will be advantageous for the population persistence. Increased variance around a suboptimal mean increases the probability of highly unfavorable years that would require survival in the persistent seed bank but also increases the probability of highly favorable years necessary for seed bank replenishment.
3. Key life‐history features that include a persistent seed bank, episodic high reproductive output and high recruitment success compensate for high dormant season mortality and short life span, making adult plant survival through the dormant season less important for population persistence.
4. Because of the key role of the seed bank in population persistence for this species in its present environment, modeling will show that population persistence is not possible without a long‐lived seed bank.
5. Modeling will also predict that artificial seed bank augmentation can substantially decrease extinction probability in at‐risk populations and that seed introduction can potentially be used to establish viable new populations of this endangered species in suitable unoccupied habitat.

## 2 METHODS

### 2.1 Study species description

*Astragalus holmgreniorum* is restricted in its current distribution to an area within 15 km of a rapidly expanding urban center, St. George, Utah, USA, at the northeastern edge of the Mojave Desert (US Fish and Wildlife Service, 2006). The species was first described by Barneby (1980) and was listed as federally endangered in 2001 (US Fish and Wildlife Service, 2001). Its narrow geographic range, habitat specialization, and locally low abundance characterize it as a rare species at apparently high risk of extinction (Rabinowitz, 1981). The species is almost completely confined to the Virgin Limestone member of the Triassic Moenkopi formation, where it occupies wash skirts and adjacent swales with relatively fine‐textured soils at the base of rocky hillslopes that generate surface runoff (US Fish and Wildlife Service, 2006; Van Buren & Harper, 2003). This overland flow provides supplemental water that permits high recruitment success and high reproductive output in favorable years, but that creates the hazard of pod dispersal into the unfavorable habitat of active washes. The leathery, partially dehiscent pods of this species are adapted for seed dispersal by the wind on a local scale, but open immediately during summer rain and release their seeds, preventing this potential seed loss (Figure 1; Houghton et al., 2020). The species is facultatively autogamous but has much higher reproductive success with pollinator‐assisted selfing (geitonogamy) and especially with outcrossing (Tepedino, 2005). The principal pollinators are large ground‐nesting native bees of the genus *Anthophora*; there is no evidence for pollen limitation under field conditions (Tepedino, 2005).

*Astragalus holmgreniorum* has phenology typical of a spring ephemeral hemicryptophyte (Rominger et al., 2019). Plants emerge as seedlings or returning adults in very early spring, grow actively through the spring, and adults complete flowering and seed production by early summer (Figure 1). All actively growing plants then enter summer dormancy; all aboveground tissues senesce, leaving only dormant shoot meristems at or slightly below the soil surface. Surviving plants remain dormant through summer, autumn, and winter, then reinitiate shoot growth in early spring the following year. As first‐year recruits rarely if ever grow large enough to produce significant quantities of seeds, plants must survive at least one dormant season in order to contribute seeds to the next generation (Rominger et al., 2019; K. R. Rominger, unpublished data).

### 2.2 Long‐term demographic study

The demographic data for this analysis were collected over the period 1991 through 2012 at two closely adjacent study sites in the State Line population of *A*. *holmgreniorum*. From 1991 to 1999 data were collected at the Demography study site (37.018969N, 113.634275W), and from 1999 to 2012 data were collected at the Atkinville Wash study site (37.022613N, 113.638133W), a few hundred meters to the west (Van Buren & Harper, 2003). The study site location change was necessitated by severe disturbance at the original site. The basic approach for the demographic study involved tagging and collecting data on all new individuals (adults and recruits) and recording the presence, plant diameter, and reproductive status (number of flowers per plant) of all previously marked individuals in a defined area (1000 m^2^ at the Demography site; 600 m^2^ at the Atkinville Wash site) at a single census date in mid to late April.

The demographic data permitted analysis either by age class or size class. We chose a stage‐based approach using plant diameter because size was a better predictor of reproductive output than plant age. Plants were measured each year and assigned to one of three size classes. Size Class 1 (S1) plants (diameter <6 cm) represented both current‐year recruits and a few older plants that had remained small or regressed in size from the previous year. Plants in this size class occasionally produced a few flowers but contributed essentially no seeds to the seed rain. Size Class 2 (S2) plants (6–15 cm diameter) and Size Class 3 (S3) plants (>15 cm diameter) were adult plants that had survived at least one dormant season and that could potentially produce seeds. For plants that survived from 1 year to the next, individuals could remain in the previous‐year size class, grow to a larger size class, or regress to a smaller size class. We calculated densities of newly recruited and adult plants on the study plot each year. We also quantified the survival probability and probability of transitioning from one size class to another from one year to the next for each size class. These data were used to calculate means and variances for each of these vital rates. See Appendix 1 for a detailed explanation.

### 2.3 Reproductive output study

We also recorded the number of flowers on each plant in the demography study each year, then calculated the proportion of plants of each reproductive size class (S2 and S3) that flowered as well as the mean number of flowers per flowering plant for each size class each year.

To translate flower number data from the demography study into an estimate of seed production, we utilized an independent reproductive output dataset (Searle, 2011). Fruit set was measured by first counting flowers, then counting mature fruits on individual marked plants in the field in 2009, a year with below‐average precipitation, and 2010, a year with above‐average precipitation. The mean number of seeds per fruit was calculated by quantifying seed number in a representative sample of intact, mature fruits. These two reproductive output parameters were then combined to obtain the mean number of seeds per flower (i.e., fruits/flower * seeds/fruit = seeds/flower) for each of the two study years for S2 and S3 plants.

The linear relationship between the number of seeds per flower for each reproductive size class and growing season precipitation in the two reproductive output study years was then used to develop a predictive equation relating seeds per flower to precipitation. The predictive equations were used to estimate seeds per flower for each year of the demographic study for S2 and S3 plants, based on the long‐term seasonal precipitation record obtained from the Prism climate interpolator (https://prism.oregonstate.edu/explorer). The mean seed production per plant each year of the demographic study based on flower number per plant for all plants of each reproductive size class could then be estimated. (See Appendix 1 for details of vital rate calculation). Seed production per unit area was estimated by multiplying by plant density each year.

We included an additional vital rate, seed rain survival, in the model to account for possible post‐dispersal losses prior to incorporation into the seed bank. We based our estimate of seed rain survival on the study of Houghton et al. (2020), which showed only small losses to seed predators or to removal from suitable habitat via overland flow. In the absence of yearly data, this vital rate was treated as time‐invariant. This parameter was included in order to avoid the assumption that all seeds produced can successfully enter and persist in the seed bank.

### 2.4 Seed bank persistence study

We used a 6‐year retrieval study with seeds of known age to examine seed bank persistence (Searle, 2011; A. Searle, unpublished data). Most of the seeds of *A*. *holmgreniorum* are physically dormant at maturity and require scarification or long‐term change in the soil to become germinable. This study showed that a constant fraction of the initial number of seeds in a cohort became nondormant each year, that is, the remaining dormant seed fraction decreased linearly through time (11.1% per year) to a value of zero at 9 years. The dormancy loss rate was insensitive to environmental variation. The fraction of nonviable ungerminated seeds in a cohort in this retrieval experiment was also insensitive to environmental variation. It increased linearly with time (3% per year) to an estimated maximum of 27% viability loss at 9 years. We used this dataset as a basis for the calculation of vital rates for seed bank transitions in the model. The seeds that become nondormant each year can either germinate within the year and potentially transition to S1 the following year, or they can remain ungerminated and enter the nondormant stage the following year (see Appendix 1 for details of study and calculations).

### 2.5 Germinant survival to census study

Germinant survival to census is an important vital rate in the model that cannot be obtained from observational data. To estimate this parameter, we used data from a recent experimental seeding (Meyer & Rominger, 2021). Seeds were acid‐scarified to render them nondormant and sown by broadcasting and raking in the field in late fall 2018 at ten introduction sites. Winter precipitation was almost twice the long‐term average in 2019–2020, and the seedings overall were highly successful, with an average return on seed (recruits/seeds planted) of 24%. All scarified seeds germinated in retrieval bags that year, making return on seed equivalent to germinant survival to census. We extrapolated from this data point to construct a regression for predicting germinant survival to census from winter precipitation and used the equation to estimate this parameter for each year of the demographic study. We could then calculate the estimated germinant survival mean and variance (see Appendix 1 for details).

### 2.6 Correlation analysis

We performed correlation analysis based on the demographic dataset using yearly precipitation data for the prior‐year summer‐fall dormant season (June–October) and for the current‐year growing season (winter: November–January, early spring: February–March, late spring: April–May) from the PRISM Data Explorer for our study site as predictors. Response variables were yearly measurements of plant survival, size class transition, and flowering. Seed output and germinant survival variables could not be included, as precipitation data were used in their estimation. We also examined whether response variables (vital rates) were correlated with each other.

### 2.7 Population matrix modeling

We adopted a stochastic discrete‐time matrix modeling approach to simulate population trends through time for *A*. *holmgreniorum* using the program MATLAB to perform simulations based on minor modifications of MATLAB code published by Morris and Doak (2002; see Appendix 2 for details of model development). This approach to stochastic population modeling uses measured or estimated variation in vital rates to introduce stochasticity.

#### 2.7.1 Defining the population matrix

We first constructed a life cycle diagram for the species life history (Figure 2) based on combinations of vital rates calculated as described in Appendix 1. The life cycle diagram corresponds directly to the transition matrix that was constructed to project population trajectories through time (Appendix 2). It consists of thirteen stages, three stages for actively growing plants in each of the three size classes, nine stages that represent dormant seeds of nine different ages in the seed bank, and one stage that represents nondormant seeds that carry over across the yearly time step. Each of the 41 matrix elements in the model reflects a transition from one stage to another and is comprised of mathematical combinations of one or more vital rates (Table 1).

**TABLE 1:** Vital rate combinations that define each of the matrix elements (stage transitions) in the life cycle diagram for *A*. *holmgreniorum* (Figure 2)

Matrix element (stage transition)	Vital rate combination^a^
Size Class 1 to Size Class 1 (Stasis)	SISURV*S1S1
Size Class 1 to Size Class 2	S1SURV*S1NOTS1*SIS2NEW
Size Class 1 to Size Class 3	SISURV*SINOTS1*S1S3NEW
Size Class 2 to Size Class 2 (Stasis)	S2SURV*S2S2
Size Class 2 to Size Class 1	(S2SURV*S2NOTS2*S2S1NEW)+ (S2RO*SRS*ND1FRAC0*GFRAC*GSURV)
Size Class 2 to Size Class 3	S2SURV*S2NOTS2*S2S3NEW
Size Class 3 to Size Class 3 (Stasis)	S3SURV*S3S3
Size Class 3 to Size Class 1	(S3SURV*S3NOTS3*S3S1NEW)+ (S3RO*SRS*ND1FRAC0*GFRAC*GSURV)
Size Class 3 to Size Class 2	S3SURV*S3NS3*S3S2NEW
Size Class 2 to Seed Bank 1	S2RO*SRS*DFRAC0
Size Class 3 to Seed Bank 1	S3RO*SRS*DFRAC0
Size Class 2 to Nondormant Stage	S2RO*SRS*ND1FRAC0*(1‐GFRAC)
Size Class 3 to Nondormant Stage	S3RO*SRS*ND1FRAC0*(1‐GFRAC)
Seed Bank 1 to Seed Bank 2	DFRAC1
Seed Bank 2 to Seed Bank 3	DFRAC2
Seed Bank 3 to Seed Bank 4	DFRAC3
Seed Bank 4 to Seed Bank 5	DFRAC4
Seed Bank 5 to Seed Bank 6	DFRAC5
Seed Bank 6 to Seed Bank 7	DFRAC6
Seed Bank 7 to Seed Bank 8	DFRAC7
Seed Bank 8 to Seed Bank 9	DFRAC8
Seed Bank 1 to Size Class 1	ND1FRAC1*GFRAC*GSURV
Seed Bank 2 to Size Class 1	ND1FRAC2*GFRAC*GSURV
Seed Bank 3 to Size Class 1	ND1FRAC3*GFRAC*GSURV
Seed Bank 4 to Size Class 1	ND1FRAC4*GFRAC*GSURV
Seed Bank 5 to Size Class 1	ND1FRAC5*GFRAC*GSURV
Seed Bank 6 to Size Class 1	ND1FRAC6*GFRAC*GSURV
Seed Bank 7 to Size Class 1	ND1FRAC7*GFRAC*GSURV
Seed Bank 8 to Size Class 1	ND1FRAC8*GFRAC*GSURV
Seed Bank 9 to Size Class 1	ND1FRAC9*GFRAC*GSURV
Seed Bank 1 to Nondormant Stage	ND1FRAC1*(1‐GFRAC)
Seed Bank 2 to Nondormant Stage	ND1FRAC2*(1‐GFRAC)
Seed Bank 3 to Nondormant Stage	ND1FRAC3*(1‐GFRAC)
Seed Bank 4 to Nondormant Stage	ND1FRAC4*(1‐GFRAC)
Seed Bank 5 to Nondormant Stage	ND1FRAC5*(1‐GFRAC)
Seed Bank 6 to Nondormant Stage	ND1FRAC6*(1‐GFRAC)
Seed Bank 7 to Nondormant Stage	ND1FRAC7*(1‐GFRAC)
Seed Bank 8 to Nondormant Stage	ND1FRAC8*(1‐GFRAC)
Seed Bank 9 to Nondormant Stage	ND1FRAC9*(1‐GFRAC)
Nondormant Stage to Nondormant Stage	(1‐GFRAC)
Nondormant Stage to Size Class 1	GFRAC*GSURV

Note

See Appendix 1 for details of vital rate definitions and calculations and Appendix 2 for transition matrix.

^a^Vital rate code explanations using examples:

Size Class 2 to Size Class 2 (Stasis): S2SURV survival probability for Size Class 2, S2S2 probability that a plant of Size Class 2 will remain in Size Class 2.

Size Class 2 to Size Class 3: S2SURV Survival probability for Size Class 2, S2NOTS2 probability than an S2 will not remain in Size Class 2, S2S3NEW probability that an S2 that does not remain in Size Class 2 will transition to Size Class 3.

Transitions from Size Class 2 or Size Class 3 to Size Class 1 include transitions among existing plants as above and also recruitment (transition to S1) from nondormant seeds produced in the current year: S3RO = seed production of Size Class 3 plants; SRS = seed rain survival; NDFRAC0 fraction of current year seeds that are nondormant; GFRAC = fraction of nondormant seeds that germinate; GSURV fraction of germinants that survive to census as S1 recruits.

The fraction of seeds produced by S2 and S3 plants that are dormant transition directly into the dormant carryover seedbank (SB1): S3RO*SRS*DFRAC0. Nondormant seeds produced in the current year can germinate and have the potential to transition to S1 plants as above, or they can fail to germinate and enter the carryover nondormant seed pool: S3RO*SRS*ND1FRAC0*(1‐GFRAC).

Dormant seeds in the seed bank that carry over each year from seeds of one age to seeds of an age 1 year older: DFRAC1 through DFRAC8

Seeds of each age in the seed bank that become nondormant can (1) germinate and potentially transition to S1 recruits or (2) enter the carryover nondormant seed pool as in:

(1) ND1FRAC1*GFRAC*GSURV

(2) ND1FRAC1*(1‐GFRAC)

Seeds in the nondormant carryover seed pool can either germinate (GFRAC) and potentially lead to S1 recruits (GSURV) or remain in the pool for another year (1‐GFRAC).

#### 2.7.2 Modeling procedure

The model starts with values for each time‐invariant vital rate and probability distributions for each variable vital rate, generated from measured means, variances, and within‐ and between‐year correlations among vital rates. It then picks vital rate values at random based on the constraints of their distributions to generate a matrix for each yearly time step in each iteration. The first time step uses an initial vector of stage values as input, and output from each matrix calculation is used as input for the next yearly time step. The model initiates each time step in May, soon after census, when seeds are mature but not yet dispersed (pre‐birth‐pulse). This is also the beginning of the dormant season, just before the last point in time when actively growing plants were present. We used our best estimates for each vital rate to create the base population matrix model. We could then simulate the effect of changes in specific vital rates on both mean stochastic growth rate (*λ*_s_ or stochastic lambda) and extinction probability.

#### 2.7.3 Extinction risk estimates

We used a cumulative distribution function to generate an extinction risk time series for each model run (Morris & Doak, 2002). Extinction risk for a given point in time into the future is defined as the proportion of model iterations in which the population falls below a quasi‐extinction threshold by that time. The quasi‐extinction threshold was conservatively but arbitrarily set at 200, that is, when numbers fell below 200 plants + seeds, the local population was considered extinct. As ca. 99% of the genets on average are present as seeds, this threshold represents a seed bank reduced to a dangerously low level. Extinction risk was positively correlated with quasi‐extinction threshold, but this relationship showed an exponential rise to maximum, with little change in extinction risk at quasi‐extinction thresholds >200 (data not shown). At very small thresholds, extinction risk was greatly reduced, but these low thresholds are not ecologically realistic.

## 3 RESULTS

### 3.1 Hypothesis 1: Population fluctuations and precipitation drivers

We used the results of the demographic study directly to evaluate our hypothesis that the effect of extreme inter‐annual variation in seasonal precipitation would be evident as extreme population fluctuations in density and seed production through time.

#### 3.1.1 Plant density and survival

**FIGURE 3:**
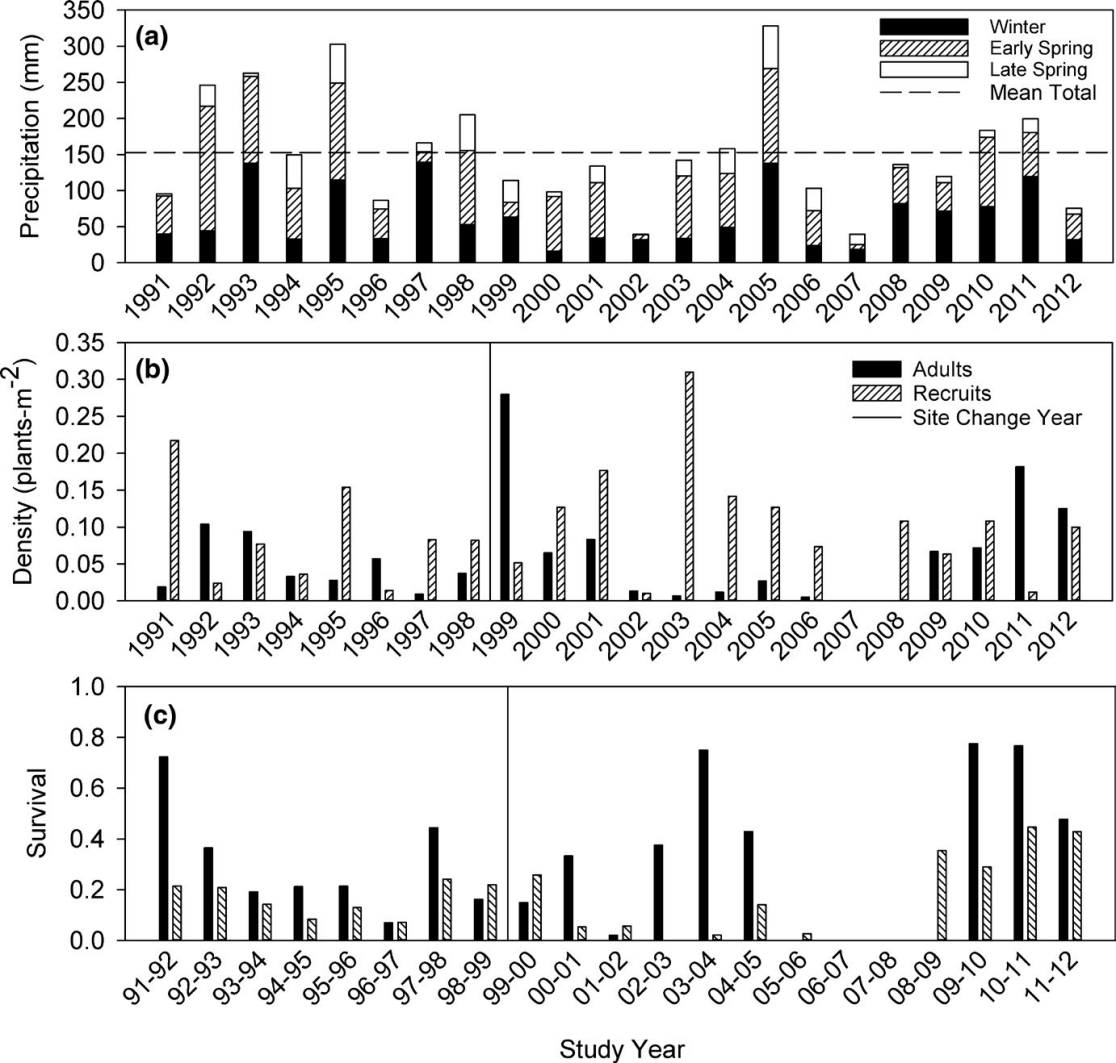
(a) Precipitation received on the study plot each year during winter (November–January), early spring (February‐March) and late spring (April‐May) (from PRISM Data Explorer: http://www.prism.oregonstate.edu/explorer/), (b) Density of adults (previously tagged returning plants alive at census in late April), and recruits (newly tagged seedlings alive at census in late April) on the demography plot each year of the study from 1991 to 2012, and (c) Proportion of individuals surviving from one yearly census to the next on the demography plot for adults and recruits

Densities of both adult plants and recruited seedlings were generally low during the 22‐year course of the demographic study (Figure 3). Adult plant density averaged 0.057 plants‐m^−2^, while the density of recruited seedlings averaged 0.095 plants‐m^−2^. Densities of both recruits and returning adults fluctuated widely, with values ranging from zero to 0.280 plants‐m^−2^ for adults and from zero to 0.310 plants‐m^−2^ for new recruits (Figure 3b). Adult plant density was higher over the first 11 years of the study, averaging 0.074 plants‐m^−2^. Starting in 2002, there was a sharp decline in adult plant density, with no adults present in 2002 and a mean density of only 0.009 plants‐m^−2^ over the period 2002–2008. Adult plants then recovered over the final 4 years of the study (2009–2012), with a mean density of 0.111 plants‐m^−2^.

Recruit density was much more stable through time than adult plant density, with some recruitment almost every year (Figure 3b). Recruitment averaged 0.095 plants‐m^−2^ over the first 11 years, 0.110 plants‐m^−2^ over the period 2002–2008, and 0.071 plants‐m^−2^ over the period 2009–2012. This suggests that conditions that permit seedling recruitment during the current season occur more frequently than conditions that permit recruited seedlings to survive through the dormant season and return as adult plants the following spring. Successful dormant season survival of 2008 recruits allowed for the recovery of adult plant density late in the study period.

During the period 2002–2008, recruit survival over the dormant season was 0.032, with zero survival in three of those years (Figure 3c). Thus, the principal reason for very low densities of adults during the period 2002–2008 was not recruitment failure, but failure of recruited seedlings to survive their first dormant season.

The mean survival of adult plants through the dormant season averaged 0.380, whereas the mean survival of recruited seedlings to adulthood was only 0.169 (Figure 3c). In spite of higher adult survival, the average life span of a plant that survived to adulthood was less than 3 years, and no individual in the study was known to survive for more than 6 years. The mean survivorship curve for this species was close to a Deevey Type II curve (Deevey, 1947), that is, a log‐linear relationship of survivorship with age, resulting in relatively constant mortality risk through time (See Appendix 3 for full discussion).

#### 3.1.2 Seed production

**FIGURE 4:**
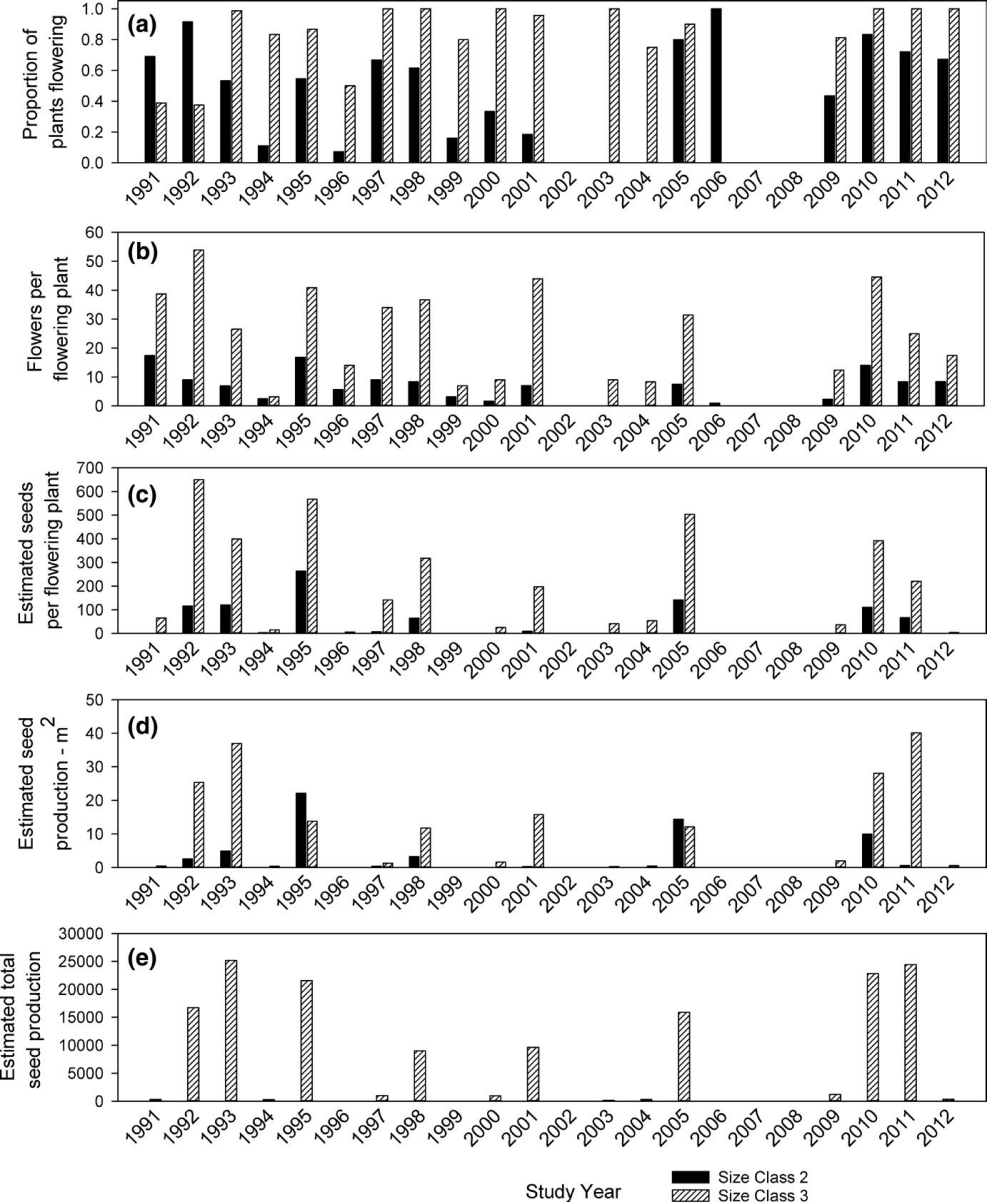
For plants of Size Class 2 (6–15 cm diameter) and Size Class 3 (>15 cm diameter): (a) Proportion of plants that flowered each census year, (b) Mean number of flowers produced by plants that flowered each census year (c) Estimated seed production per plant each census year, (d) Estimated seed production per square meter each census year (obtained by multiplying plant density by seeds per plant for each census year and size class), and (e) Total estimated seed production each census year on the 600 m^2^ study plot (based on summing seed production of S2 and S3 plants per m^2^ and multiplying by 600)

Most S3 plants flowered in the majority of years when plants were present (mean of 84% of S3 plants flowered; Figure 4a). Flowering was much more variable for S2 plants; only about half (47%) of the S2 plants flowered on average. S3 plants that flowered also produced almost six times more flowers than S2 plants, averaging 17.9 flowers per plant versus only 3.1 flowers per plant for S2 plants (Figure 4b). S2 plants also produced fewer seeds per flower than S3 plants, and this difference was more pronounced in the less favorable year (Searle, 2011, see Appendix 1). The combination of fewer flowers per plant and fewer seeds per flower for S2 plants resulted in a much smaller seed output for S2 plants even in favorable years (Figure 4c).

When we calculated seed production on an area basis, we obtained the striking result that significant seed production was highly episodic (Figure 4d). Most of the seeds produced over the 22‐year study period were produced in only a handful of years (Figure 4e). An estimated 150,000 seeds were produced in the study plots over the 22‐year period, of which 115,000 or approximately 77% were produced by S3 plants. Estimated total annual production exceeded 15,000 seeds in only 6 years, and these 6 years accounted for almost 85% of total seed production. Another 12% was accounted for by two additional years that approached production of 10,000 seeds. The remaining 14 years accounted for <3% of total production.

#### 3.1.3 Precipitation as a driver of vital rate variance

Results of the correlation analysis generally supported Hypothesis 1, namely that high variance in vital rates was driven by stochastic variation in environmental quality as reflected in inter‐annual variation in seasonal precipitation. Vital rates were generally positively correlated with winter‐spring precipitation. However, these correlations were often not very strong, even though the inter‐annual variation in precipitation was extreme (Table 2, Figure 3a). Very dry years (2002, 2007) were associated with failure to emerge successfully from dormancy and a complete lack of actively growing plants, but exceptionally high‐precipitation years (e.g., 2005) were not necessarily associated with high survival or high reproductive output (Figures 3 and 4). The pattern of positive correlation with precipitation was generally consistent even when correlation coefficients were not significant, however. Precipitation correlations associated with the growth of smaller size classes or stasis in the largest size class were all positive and mostly significant, as were all correlations associated with flowering. In contrast, correlation coefficients associated with size regression or stasis in the smaller size classes were all negatively correlated with winter‐spring precipitation, though none of these were statistically significant.

**TABLE 2:** Correlations between vital rates and precipitation drivers for the previous summer‐fall dormant period (Jun–Oct) and the winter‐spring active period (Nov–May), based on the 22‐year demographic dataset (Figures 3 and 4) and PRISM Data Explorer precipitation estimates (Figure 3a)

Population measure	Winter–spring precipitation		Summer‐fall precipitation	
	Correlation coefficient	*p*‐Value	Correlation coefficient	*p*‐Value
*Survival*				
S1 Survival	0.2909	.201	−0.0726	.754
Adult survival (S2 and S3)	0.0150	.949	−0.4838	.026
S2 Survival	−0.0520	823	−0.5715	.007
S3 Survival	0.1325	.567	−0.3694	.099
*Flowering*				
S2 Proportion flowering	0.5481	.010	0.2282	.320
S3 Proportion flowering	0.5116	.018	0.1023	.659
S2 Flowers per plant	0.5399	.012	0.1171	.613
S3 Flowers per plant	0.6492	.002	0.1204	.603
*Size class transitions*				
S1 to S1 (Stasis)	−0.2896	.203	−0.0132	.955
S1 to S2 (Growth)	0.1881	.414	−0.2009	.383
S1 to S3 (Growth)	0.4981	.022	0.1916	.405
S2 to S1 (Regression)	−0.4033	.070	0.0121	.959
S2 to S2 (Stasis)	−0.0711	.759	0.1454	.529
S2 to S3 (Growth)	0.6380	.002	−0.1074	.643
S3 to S1 (Regression)	−0.2596	.256	−0.1970	.392
S3 to S2 (Regression)	−0.2159	.347	−0.2228	.332
S3 to S3 (Stasis)	0.5569	.009	0.2082	.365

Note

Probabilities shaded yellow are significant at *p* < .05; probabilities shaded peach are marginally significant at *p* > .05 but *p* < .10.

Precipitation during the summer‐fall dormant period had no strong or consistent effect on any vital rates except those associated with adult survival over the dormant season (Table 2). Surprisingly, the significant effect of dormant season precipitation on adult survival was negative, that is, plants suffered higher mortality with increased warm‐season precipitation. The reason for this is not known.

Correlations among vital rates confirmed the patterns seen for winter‐spring precipitation. Over‐dormant season survival was positively correlated among size classes and these correlation coefficients were significant or marginally significant (Table 3). Survival was consistently positively correlated with flowering and usually also positively associated with growth. Significant correlations among size class transitions were generally positive for pairs of transitions that indicated growth, whereas negative correlations were found between pairs of transitions for different size classes that indicated growth versus size regression. Stasis in S1 was positively correlated with size regression for larger size classes, whereas stasis in S3 was positively correlated with growth for smaller size classes. The highest positive correlations observed among vital rates were those associated with flowering. These vital rates were also positively correlated with positive growth transitions for smaller size classes and stasis for S3 plants. Results indicate that high inter‐annual variation in seasonal precipitation is likely the principal driver of stochastic vital rate variation. They also suggest that the precipitation regimes conducive to growth and flowering are similar.

**TABLE 3:** Correlations among *Astragalus holmgreniorum* vital rates calculated from 22 years of demographic data (S1SURV, S2SURV, S3SURV=over‐dormant season survival for size classes S1, S2, and S3; S2FLPROP, S3FLPROP, S2FLPLT, S3 FLPLT = proportion of plants flowering and flowers per flowering plant for S2 and S3; remaining nine columns represent size class transitions among S1, S2, and S3)

	S1 SURV	S2 SURV	S3 SURV	S2 FL PROP	S3 FL PROP	S2 FL PLT	S3 FL PLT	S1S1	S1S2	S1S3	S2S1	S2S2	S2S3	S3S1	S3S2	S3S3
S1SURV	1.000	0.367	0.404	0.314	0.253	0.391	0.337	−0.307	0.185	0.156	−0.087	−0.192	0.183	−0.172	−0.020	0.234
		0.102	0.070	0.166	0.269	0.080	0.135	0.188	0.434	0.510	0.715	0.419	0.440	0.469	0.935	0.321
S2SURV	0.367	1.000	0.548	0.121	0.104	0.188	0.209	0.088	0.351	−0.041	0.286	−0.191	0.240	−0.174	0.192	0.136
	0.102		0.010	0.600	0.655	0.413	0.362	0.712	0.129	0.865	0.221	0.419	0.307	0.463	0.418	0.568
S3SURV	0.404	0.548	1.000	0.209	0.390	0.305	0.253	−0.449	0.611	0.303	−0.263	−0.071	0.302	−0.088	0.022	0.026
	0.070	0.010		0.364	0.080	0.179	0.268	0.047	0.004	0.193	0.263	0.766	0.196	0.714	0.927	0.914
S2 FL PROP	0.314	0.121	0.209	1.000	0.179	0.618	0.627	0.027	0.179	0.243	−0.293	−0.045	0.274	−0.260	−0.065	0.438
	0.166	0.600	0.364		0.439	0.003	0.002	0.912	0.450	0.302	0.210	0.850	0.243	0.269	0.787	0.053
S3 FL PROP	0.253	0.104	0.390	0.179	1.000	0.338	0.448	−0.418	0.723	0.502	−0.298	0.211	0.661	−0.093	0.157	0.558
	0.269	0.655	0.080	0.439		0.134	0.042	0.066	0.0003	0.024	0.202	0.372	0.002	0.696	0.508	0.011
S2 FL PLT	0.391	0.188	0.305	0.618	0.338	1.000	0.849	−0.098	0.200	0.358	−0.247	0.056	0.557	−0.005	0.062	0.390
	0.080	0.413	0.179	0.003	0.134		<.0001	0.681	0.398	0.122	0.293	0.815	0.011	0.983	0.796	0.089
S3 FL PLT	0.337	0.209	0.253	0.627	0.448	0.849	1.000	−0.266	0.335	0.468	−0.306	0.039	0.642	−0.129	−0.037	0.632
	0.135	0.362	0.268	0.002	0.042	<.0001		0.257	0.148	0.038	0.190	0.871	0.002	0.587	0.877	0.003
S1S1	−0.307	0.088	−0.449	0.027	−0.418	−0.098	−0.266	1.000	−0.459	−0.478	0.475	−0.025	−0.261	0.215	−0.014	−0.376
	0.188	0.712	0.047	0.912	0.066	0.681	0.257		0.036	0.029	0.030	0.916	0.254	0.349	0.953	0.093
S1S2	0.185	0.351	0.611	0.179	0.723	0.200	0.335	−0.459	1.000	−0.017	−0.184	0.294	0.346	0.052	0.410	0.386
	0.434	0.129	0.004	0.450	0.0003	0.398	0.148	0.036		0.942	0.426	0.196	0.124	0.823	0.065	0.084
S1S3	0.156	−0.041	0.303	0.243	0.502	0.358	0.468	−0.478	−0.017	1.000	−0.269	−0.014	0.380	−0.240	−0.269	0.445
	0.510	0.865	0.193	0.302	0.024	0.122	0.038	0.029	0.942		0.238	0.953	0.089	0.295	0.238	0.043
S2S1	−0.087	0.286	−0.263	−0.293	−0.298	−0.247	−0.306	0.475	−0.184	−0.269	1.000	−0.048	−0.423	−0.050	0.037	−0.345
	0.715	0.221	0.263	0.210	0.202	0.293	0.190	0.030	0.426	0.238		0.837	0.056	0.831	0.874	0.125
S2S2	−0.192	−0.191	−0.071	−0.045	0.211	0.056	0.039	−0.025	0.294	−0.014	−0.048	1.000	−0.307	0.669	0.670	0.053
	0.419	0.419	0.766	0.850	0.372	0.815	0.871	0.916	0.196	0.953	0.837		0.175	0.001	0.001	0.820
S2S3	0.183	0.240	0.302	0.274	0.661	0.557	0.642	−0.261	0.346	0.380	−0.423	−0.307	1.000	−0.294	−0.234	0.642
	0.440	0.307	0.196	0.243	0.002	0.011	0.002	0.254	0.124	0.089	0.056	0.175		0.196	0.308	0.002
S3S1	−0.172	−0.174	−0.088	−0.260	−0.093	−0.005	−0.129	0.215	0.052	−0.240	−0.050	0.669	−0.294	1.000	0.472	−0.278
	0.469	0.463	0.714	0.269	0.696	0.983	0.587	0.349	0.823	0.295	0.831	0.001	0.196		0.031	0.223
S3S2	−0.020	0.192	0.022	−0.065	0.157	0.062	−0.037	−0.014	0.410	−0.269	0.037	0.670	−0.234	0.472	1.000	−0.141
	0.935	0.418	0.927	0.787	0.508	0.796	0.877	0.953	0.065	0.238	0.874	0.001	0.308	0.031		0.542
S3S3	0.234	0.136	0.026	0.438	0.558	0.390	0.632	−0.376	0.386	0.445	−0.345	0.053	0.642	−0.278	−0.141	1.000
	0.321	0.568	0.914	0.053	0.011	0.089	0.003	0.093	0.084	0.043	0.125	0.820	0.002	0.223	0.542	

Note

Gray‐shaded rows show correlation coefficients, white rows show probabilities of significance. *p*‐values shaded yellow are significant at *p *< .01; *p*‐values shaded peach are marginally significant at *p *> .05 but *p *< .10.

### 3.2 Hypothesis 2: The effect of environmental stochasticity

Best estimates for vital rates resulted in a base model for *A*. *holmgreniorum* with a mean stochastic growth rate (*λ*_s_) of 1.0942 ± 1.242 (mean ± standard deviation; Table 4). This indicates that, on average, this model population of *A*. *holmgreniorum* can be expected to grow through time. However, mean *λ*_s_ > 1 was associated with a very large standard deviation due to high variance in vital rates, which in turn is a result of extreme inter‐annual variation in year quality (Figure 3a). The population represented by this version of the model has a 47% chance of extinction (i.e., dropping below the 200‐individual quasi‐extinction threshold) within 50 years, assuming that the conditions that generated its vital rates in the past continue into the future. This extinction risk is based on the likelihood that a chance series of unfavorable years will prevent seed bank replenishment and lead to steep population decline some time during the 50‐year period. Even though mean *λ*_s_ is >1, there is still a relatively high probability of extinction for this small population.

**TABLE 4:** Values for the model parameters *λ*_d_ (deterministic growth rate) and mean and standard deviation of *λ*_s_ (stochastic growth rate) for the base population viability model for *A*. *holmgreniorum* (2001 initial vector of stage values) and for models testing the sensitivity of model parameters to variation in vital rates that measure survival of actively growing plants

Vital rate	Change from base value	*λ*_d_	Mean *λ*_s_	SD *λ*_s_ ^a^
Base model	0	0.9485	1.0942	1.242
Germinant survival^b^	−0.5	0.8657	0.9974	1.237
	−0.1	0.9349	1.0757	1.250
	+0.1	0.9611	1.1045	1.254
	+0.5	1.0098	1.1566	1.250
S1 Survival	−0.5	0.8620	1.0410	1.281
	−0.1	0.9341	1.0862	1.245
	+0.1	0.9620	1.1210	1.247
	+0.5	1.0093	1.1743	1.219
S2 Survival	−0.5	0.9187	1.0892	1.244
	−0.1	0.9426	1.0923	1.245
	+0.1	0.9542	1.0950	1.244
	+0.5	0.9767	1.1042	1.250
S3 Survival	−0.5	0.9312	1.0780	1.242
	−0.1	0.9448	1.0805	1.243
	+0.1	0.9523	1.0905	1.250
	+0.5	0.9694	1.1082	1.242

^a^ Standard deviations represent back‐transformed standard deviations for log‐lambda and are therefore not symmetrically distributed around the mean.

^b^ Changes in model parameter values for germinant survival were derived by changing the estimated maximum germinant survival fraction by the indicated proportion and then calculating the resulting mean value across all years. See Appendix 1 for full explanation.

The large standard deviation around mean *λ*_s_ makes it difficult to project the model population into the future with any certainty and thus to predict its probability of extinction in concrete terms. However, the deterministic version of the model_,_ which is based on vital rate mean values (standard deviation zero), resulted in a *λ*_d_ of 0.9435 and virtually certain extinction (Table 4). This indicates that the level of stochasticity reflected in measured vital rate variances resulted in a dramatically higher probability of population persistence than in the deterministic version, even though this stochasticity creates uncertainty in predicting whether or when the population will become extinct. This result supports Hypothesis 2: Given the low quality of an average year in its warm desert habitat, this population requires a stochastically varying environment to persist.

### 3.3 Hypothesis 3: Compensating for low dormant season survival

**FIGURE 5:**
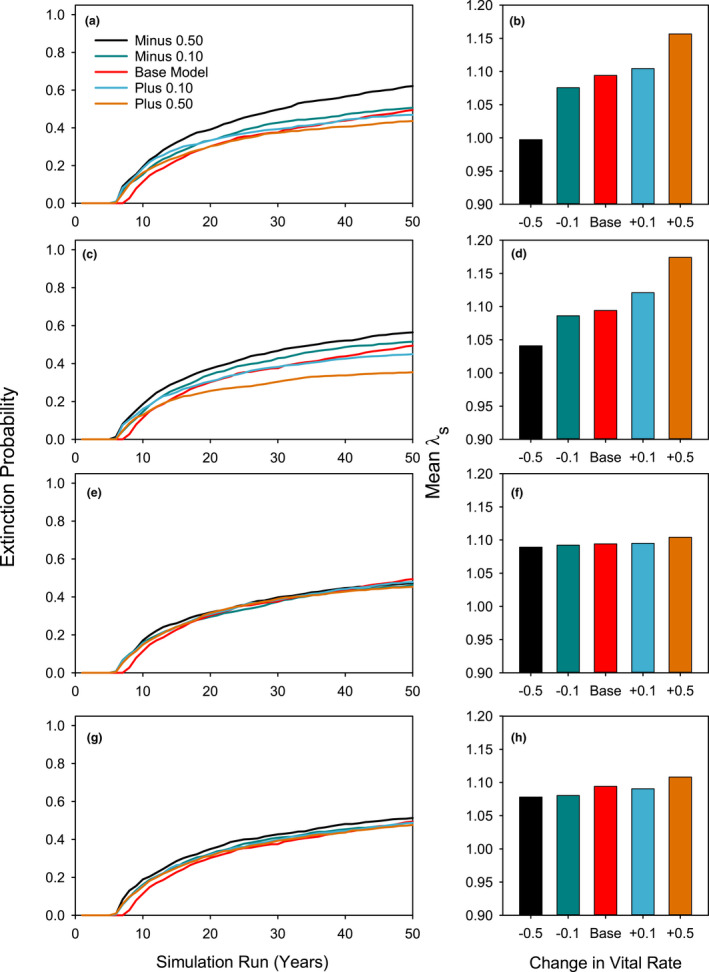
Effects on cumulative 50‐year extinction risk and mean *λ*_s_ of changing survival vital rates by increasing or decreasing the value in the base model by 0.1 or 0.5 for germinant survival to census (a, b), survival of Size Class 1 plants (c, d), survival of Size Class 2 plants (e, f), and survival of Size Class 3 plants (g, h)

Examining the effect of changing vital rate means on model outcomes is a form of sensitivity analysis for stochastic population modeling that helps to identify which vital rates are most important in determining population trajectories. We proposed in Hypothesis 3 that low dormant‐season survival is a given in the life history of this species, and that other stages of the life cycle would be able to compensate for this low survival and thus reduce its importance. To test this hypothesis, we reduced or increased survival vital rates of germinants, S1, S2, or S3 plants by 10% or 50%. As predicted, these changes in survival for S2 and S3 adult plants had negligible impact on mean *λ*_s_ (Table 4, Figure 5f,h) and also had minimal effect on cumulative extinction risk (Figure 5e,g). When S1 survival, which includes dormant season survival of S1 recruits, was changed, mean *λ*_s_ and extinction risks showed more sizeable changes (Figure 5c,d). These changes were even more evident when germinant survival to census was manipulated (Figure 5a,b). Germinant survival was the only case where reducing survival by 50% resulted in *λ*_s_ < 1. These results support the hypothesis that the population growth rate is insensitive to reductions in survival for adult plants, but is much more sensitive to survival reductions for germinants and new recruits.

**FIGURE 6:**
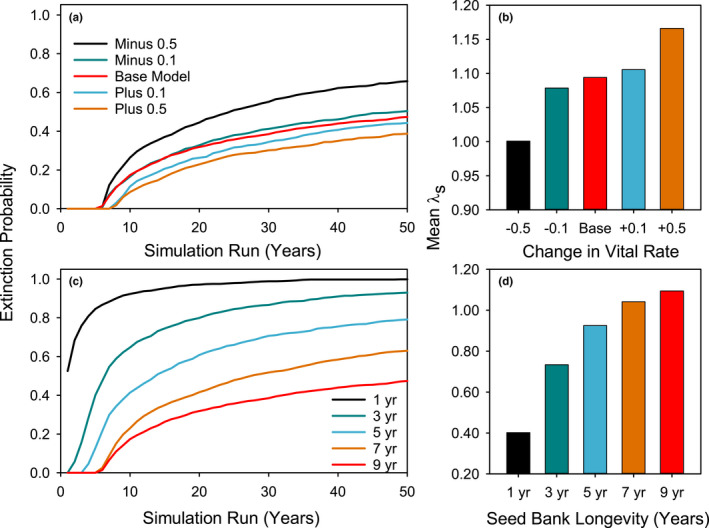
Effect on cumulative 50‐year extinction risk and mean *λ*_s_ of changing reproductive output vital rates (S2 and S3 plants combined) by 0.1 or 0.5 (a, b) or decreasing maximum seed bank longevity from 9 years to 1 year (c, d). Note scale change for mean *λ*_s_ axis in panel d

We hypothesized that high reproductive output would be a key adaptation for the survival of this species in a warm desert environment and that seed production per plant would thus be important in determining model outcomes. The sensitivity analysis also supported this hypothesis (Table 5, Figure 6a,b), with a strong effect of 50% reduction similar to the effect of 50% reduction in germinant survival.

**TABLE 5:** Values for the model parameters *λ*_d_ (deterministic growth rate) and mean and standard deviation of *λ*_s_ (stochastic growth rate) for the base population viability model for *A*. *holmgreniorum* (2001 initial vector of stage values) and for models testing the sensitivity of model parameters to variation in vital rates that measure seed production and seed bank longevity

Vital rate	Change from base value	*λ*_d_	Mean *λ*_s_	SD *λ*_s_ ^a^
Base model	0	0.9435	1.0942	1.242
Seed production	−0.5	0.8658	1.0009	1.268
	−0.1	0.9349	1.0786	1.247
	+0.1	0.9611	1.1057	1.234
	+0.5	1.0047	1.1661	1.232

	Duration	*λ*_d_	Mean *λ*_s_	SD *λ*_s_ ^a^
Seed bank longevity	9 years (Base)	0.9435	1.0942	1.242
	7 years	0.9521	1.0413	1.293
	5 years	0.9564	0.9253	1.384
	3 years	0.9615	0.7337	1.477
	1 year	0.9699	0.4020	1.650

^a^ Standard deviations represent back‐transformed standard deviations for log‐lambda and are therefore not symmetrically distributed around the mean.

The sensitivity analysis showed parallel changes in *λ*_d_ when survival and reproductive vital rates were manipulated, but even with 50% increases, *λ*_d_ only approached or barely exceeded 1.00 for any of these vital rates (Tables 4 and 5).

### 3.4 Hypothesis 4: Importance of the seed bank for population persistence

The most dramatic effects on model behavior were obtained by manipulating the vital rates that determine maximum seed longevity (Table 5, Figure 6c,d). As outlined in Hypothesis 4, we expected that a long‐lived seed bank would be necessary for population persistence in the stochastic warm desert environment, where harsh conditions in some years preclude the presence of actively growing plants (Figure 3). To test this hypothesis, we modified the fraction of seeds expected to remain dormant from 1 year to the next (as well as the corresponding fraction expected to become nondormant) in the transitions from stages SB1 through SB9 to reflect shortened seed persistence periods of seven, five, three, one, and zero years. We did not change viability loss rates, but instead limited seed bank persistence by increasing the fraction transitioning from dormant to nondormant each year (see Appendix 1 for full explanation).

When seed bank maximum persistence was shortened from 9 years to 7 years, mean *λ*_s_ dropped to 1.0413 and the extinction probability at 50 years increased to 0.619 (Table 5, Figure 6d). For 5‐year maximum persistence, mean *λ*_s_ was reduced to 0.9253 and 50‐year extinction risk increased to 0.774, while for 3‐year maximum persistence, mean *λ*_s_ reached a low of 0.7337, with 0.922 probability of extinction within 50 years. With a maximum persistence of one year, mean *λ*_s_ dropped to 0.4020 and 50‐year extinction became virtually certain, while a model with no seed bank carryover, that is, with all seeds becoming nondormant the year of their production, resulted in essentially immediate extinction. In contrast, reductions in seed bank longevity increased *λ*_d_, although these increases were small, and *λ*_d_ never exceeded 1.0. These increases are consistent with theory that predicts that delaying reproduction should reduce *λ*_d_, that is, in a constant environment, early reproduction is always favored. With a value of 5 years or less for seed bank persistence, *λ*_d_ > *λ*_s_, also as predicted by theory (Table 5; Tuljapurkar & Orzack, 1980). These results strongly support the hypothesis that a persistent seed bank is essential to population persistence in *A*. *holmgreniorum*, and also that the advantages of stochasticity for population persistence cannot be realized without the presence of a persistent seed bank.

### 3.5 Hypothesis 5: Seed addition effects on extinction risk

We performed the base model using the initial stage vector from 2001, a year with a somewhat above the average total number of individuals, including actively growing plants. We first tested whether a change in initial stage vector values would substantially change extinction risk relative to the base model. We substituted the initial stage vector for 2007, a year with a below‐average total number of individuals and no actively growing plants. This change had a substantial effect, raising 50‐year extinction risk from 47% to 60% (Table 6).

**TABLE 6:** Effects of changes in the initial vector of stage values on 50‐year quasi‐extinction risk, including the effect of: (1) using initial vectors of stage values for different years from the field data, (2) augmenting populations by adding seeds, (3) introducing new populations by planting seeds and/or mature plants into suitable unoccupied habitat, and (4) considering the effects of population size (area)

Initial vector of stage values	50‐Year extinction risk
Base model (2001 Start year)	0.474
Alternate start year (2007)	0.599
*Augmentation*	
2001 + 2000 SB1	0.450
2001 + 5000 SB1	0.440
2007 + 2000 SB1	0.555
2007 + 5000 SB1	0.525
*Introduction*	
50 S3	0.652
2000 SB1	0.642
5000 SB1	0.547
10,000 SB1	0.516
10,000 SB1 + 50 S3	0.457
*Population size*	
Base Model × 10 (0.6 ha)	0.355
Base Model × 100 (6.0 ha)	0.272
Base Model × 1000 (60 ha)	0.194

The importance of the seed bank in population persistence coupled with the result above suggested that population augmentation through the addition of seeds could substantially lower extinction risk. Changing the vector of initial stages in the 2001 base model by adding 2000 or 5000 seeds to SB1 had a small effect (47% to 45% and 44% 50‐year extinction risk, respectively), while similar changes to the vector of initial stages for the 2007 model lowered the 50‐year extinction risk from 60% to 56% and 52% (Table 6). This supported the hypothesis that population augmentation through seed addition could potentially reduce extinction risk.

We also tested the hypothesis that new populations in unoccupied suitable habitat could be initiated through seed introduction. Adding 2000 seeds to SB1 with other stages set at zero generated resulted in a model with 64% 50‐year extinction risk, similar to a model with the addition of 50 reproductive S3 plants (65% extinction risk; Table 6). Increasing seed addition further reduced extinction risk (5000‐seed addition, extinction risk 0.547; 10,000‐seed addition, 0.516 extinction risk). Finally, combining a 10,000‐seed starting population in SB1 with successful early‐spring transplanting of 50 reproductive S3 individuals lowered 50‐year extinction risk to 46%, a result similar to the 2001 base model.

## 4 DISCUSSION

### 4.1 Environmental drivers of inter‐annual vital rate variation

Our first hypothesis was that stochastic inter‐annual variability in seasonal precipitation is a principal driver of high variance in vital rates. This was supported by a strong trend overall for positive correlations of vital rates related to survival, growth, and flowering with winter‐spring precipitation over the 22 years of the study, even though many individual correlation coefficients were not statistically significant (Table 2). The relatively weak correlation with seasonal precipitation suggests that more subtle differences in rainfall periodicity, combined with other factors that influence vital rates, must act to obscure the more obvious effects of inter‐annual variation in precipitation. Efforts to correlate specific vital rates with precipitation during key time periods within the season did not result in any appreciable improvement in correlation coefficients, however. Very small sample size in some years resulted in imprecise vital rate estimates that could negatively impact correlation analysis. Also, vital rates do not necessarily vary linearly with precipitation, which would also tend to lower correlation coefficients. Consistent positive correlations among vital rates associated with survival, growth, and flowering lent support to the hypothesis that they were responding to the same precipitation drivers. We found no evidence for significant temporal autocorrelation in either precipitation drivers or vital rates, indicating that correlations among them are more likely to reflect direct or indirect causal relationships.

### 4.2 The role of environmental stochasticity

Our second hypothesis was that, given the mean current conditions that result in *λ*_d_ < 1, environmental stochasticity is necessary for the survival of this species. This hypothesis was supported. The deterministic version of the base model, with vital rates fixed at mean values, resulted in eventual near‐certain extinction (*λ*_d_ = 0.9485). Values for mean *λ*_s_ were almost always much higher than those for *λ*_d_ (Tables 4 and 5). The value of mean *λ*_s_ approached and became smaller than *λ*_d_ only when seed longevity was drastically reduced, showing that the persistent seed bank is necessary for the advantages of stochasticity for this species to be realized (Table 5).

Population persistence depends on favorable year sequences for episodic replenishment of the persistent seed bank, which in turn buffers against extinction during unfavorable year sequences that prevent adult plant survival and seed production. Thus, if every year were average, this species would become extinct. The positive effect of stochasticity in this species may be a consequence of convex relationships between important vital rates such as reproductive output with precipitation, a key environmental driver in warm deserts. The buffering effect of an environmentally insensitive seed dormancy loss rate could further reinforce the positive effect of stochasticity in this system (Lawson et al., 2015).

### 4.3 Life‐history strategy and survival in the warm desert

Our third hypothesis was that survival for *A*. *holmgreniorum* in the warm desert environment would hinge on adaptations that could compensate for low dormant‐season survival of adult plants, thus rendering adult dormant‐season survival relatively unimportant for population persistence. This hypothesis was supported. Reductions of 50% in mean survival of S2 and S3 plants (i.e., after at least one opportunity for seed production) had no appreciable effect on mean *λ*_s,_ while a reduction in similar magnitude in reproductive output had a much larger effect (Table 3). Reducing either recruit survival or dormant season survival of S1 plants by 50% had a notably more negative impact than the same survival reduction for post‐reproductive plants.

### 4.4 Importance of the persistent seed bank

Our fourth hypothesis, that the persistent seed bank would be essential for population persistence in this species, was strongly supported. Reductions in the number of years that seeds could persist in the seed bank had a dramatic negative effect on mean *λ*_s_ as well as on extinction risk (Table 5, Figure 5c,d). This shows that without a persistent seed bank, the chances of long‐term survival in the warm desert for a species with the spring‐ephemeral hemicryptophyte life form would be near zero. Episodic high seed production, high recruitment success, and the capacity to form a long‐lived seed bank are key features of the warm desert‐adapted life‐history strategy that enables long‐term persistence of *A*. *holmgreniorum* in spite of its short life span.

The coupling of higher reproductive output with a shorter life span in comparison to the life histories of perennial spring ephemerals of more mesic habitats is an expression of a more r‐selected or “fast” life‐history strategy (Salguero‐Gómez, 2017). Longer‐lived spring ephemerals include deciduous forest understory species (Augspurger & Salk, 2017; Lapointe, 2001) as well as some species of steppe habitats (e.g., *Astragalus scaphoides*; Gremer & Sala, 2013). The emphasis on a persistent seed bank in *A*. *holmgreniorum* makes its life‐history strategy even more similar to that of desert annual species, even though it does not produce seeds in the first year of life. Its short life span is presumably imposed by the stresses it experiences as an adult, rather than being genetically programmed into its life cycle as it is for annual plants (Figure 4b).

### 4.5 Seed addition as a conservation strategy

Our fifth hypothesis was that the model would demonstrate that seed addition could potentially be an effective conservation strategy for this endangered species. This hypothesis was supported. The model demonstrated high sensitivity of extinction risk to the vector of initial stage values, including increases to the seed bank. It makes intuitive sense that a larger starting population would have reduced extinction risk in a given time period. For a short‐lived perennial in the highly stochastic warm desert environment, where plant numbers vary widely between years, this led to widely divergent predictions of extinction risk depending on which year was chosen to provide starting values. There appears to be a moving window of extinction risk through time, with the population passing through periods of lower and higher extinction risk as population numbers and stage distributions fluctuate. We used the initial vector of stages in the base model as a standard for detecting the effect of variation in other model parameters on both mean *λ*_s_ and extinction risk, but this does not imply that the base model extinction risk time course is more likely than an extinction curve generated using a different initial vector of stages. The value of mean *λ*_s_ was largely insensitive to the vector of initial stage values as predicted by theory (Caswell, 2001).

Our model has shown that seed addition can substantially reduce extinction risk, supporting the idea of augmentation of at‐risk populations through seeding. Introducing seeds into unoccupied habitat was shown to have the potential to create populations that could be persistent, creating the possibility of establishing new populations through seed introduction.

The model has also provided some useful insights into other ways that extinction risk for this species might be reduced through management. The importance of high reproductive output for population persistence has drawn attention to the need to protect and enhance habitat for the ground‐nesting bees that are its principal pollinators (Tepedino, 2005). The importance of high recruitment success highlights the need to manage the increasing threat from invasive winter annual grasses that can outcompete *A*. *holmgreniorum* seedlings in the spring. And the importance of dormant season survival of recruits prompted a recent study documenting the negative impact of cattle trampling on recruit survival (Searle & Meyer, 2020).

## 5 CONCLUSIONS

Our population matrix model for *A*. *holmgreniorum* using best estimates of vital rates and an initial vector of stage values based on field data resulted in mean *λ*_s_ > 1, indicating that this population of *A*. *holmgreniorum* is predicted on average to grow if past environmental conditions persist into the future. However, extreme inter‐annual variation in environmental quality resulted in very large variances in vital rates, generating a substantial risk of local extinction even when mean *λ*_s_ > 1. Our model permitted meaningful tests of hypotheses about life‐history strategy. We determined that the species depends on a stochastic environment for population persistence, but that stochasticity only confers this advantage if there is a persistent seed bank to permit survival through unfavorable years. We confirmed that the species compensates for high adult mortality during the dormant season by employing a strategy further along the “fast‐slow” continuum that emphasizes high seed production in the first years of adult life and high recruit survival, particularly in more favorable years. We found that large inter‐annual variation in year quality related to precipitation generally resulted in very large variances in vital rates. The wide variance associated with mean *λ*_s_ as well as the impact of the initial vector of stage values on extinction risk showed that extinction risk is not predictable in this highly variable environment. This led to the concept of a moving window of extinction risk through time, with higher risk during periods of lower abundance.

The strong effect of the initial stage vector on extinction risk also served as a reminder that the extinction risk evaluated in this analysis is for only a small subset of the total number of individuals of this species measured over a very small area. Given the same life‐history strategy and the same stochastic environment, the extinction risk to the species as a whole or even to the larger population that was subsampled for this study would be far lower (Table 6).

Simulations showing the possible effectiveness of seed addition or introduction may provide a novel management strategy for reducing extinction risk. Following recommendations based on our modeling effort, augmentation and introduction projects have already been successfully implemented for this species (Meyer & Rominger, 2021) using salvaged seeds as well as seeds produced in cultivation (Schultz et al., 2021).

## CONFLICT OF INTEREST

The authors declare no conflict of interest.

## AUTHOR CONTRIBUTIONS

**Renee Van Buren:** Conceptualization (lead); Data curation (lead); Formal analysis (supporting); Funding acquisition (lead); Investigation (lead); Methodology (supporting); Project administration (lead); Validation (supporting); Visualization (supporting); Writing‐original draft (supporting); Writing‐review & editing (supporting). **Alyson B. Searle:** Data curation (supporting); Investigation (supporting); Methodology (supporting); Writing‐original draft (supporting); Writing‐review & editing (supporting). **Susan E. Meyer:** Conceptualization (equal); Formal analysis (lead); Funding acquisition (supporting); Investigation (equal); Methodology (equal); Validation (lead); Visualization (lead); Writing‐original draft (lead); Writing‐review & editing (lead).

## Figures and Tables

**FIGURE A1.1:**
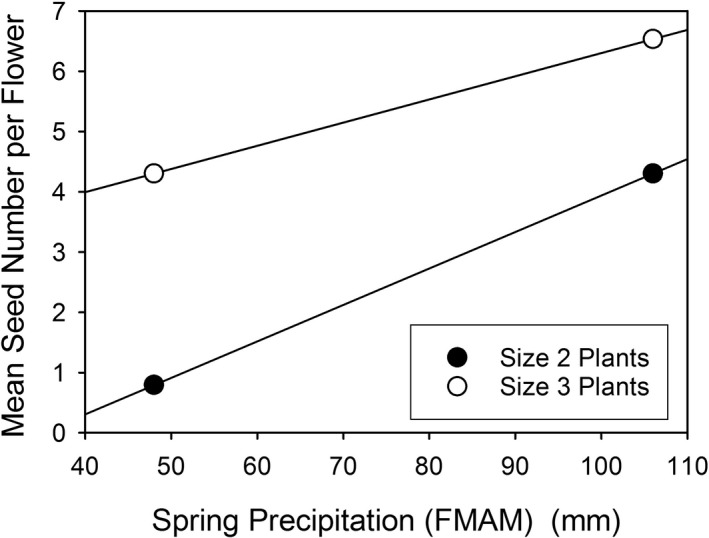
Plotted regression equations relating seed number per flower for S2 and S3 plants to spring precipitation in 2009 (48 mm) and 2010 (106 mm) (modified from Searle, 2011)

**FIGURE A1.2:**
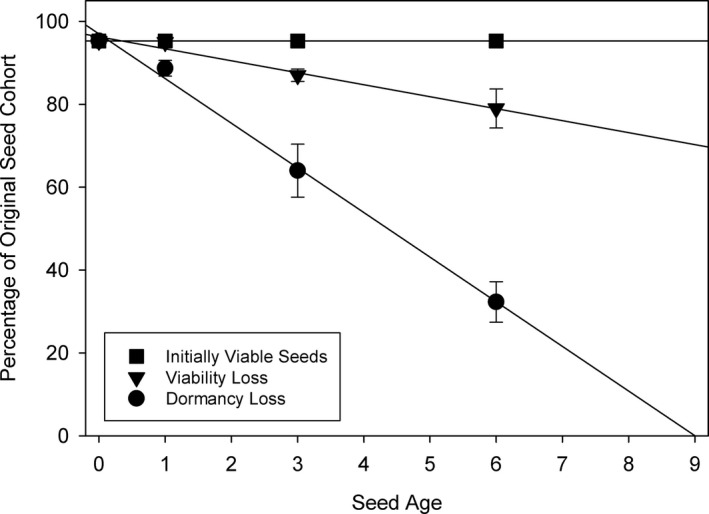
Results of a seed retrieval experiment showing initial viability, viability loss, and dormancy loss over 6 years, with values extrapolated to 9 years, the projected maximum time for dormant seeds of a cohort to persist in the seed bank (modified from Searle, 2011)

**FIGURE A1.3:**
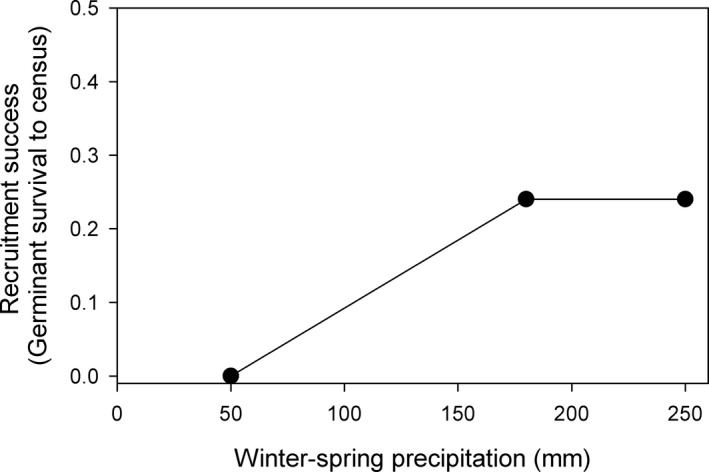
Schematic relationship between winter‐spring precipitation and germinant survival to census

**FIGURE A3.1:**
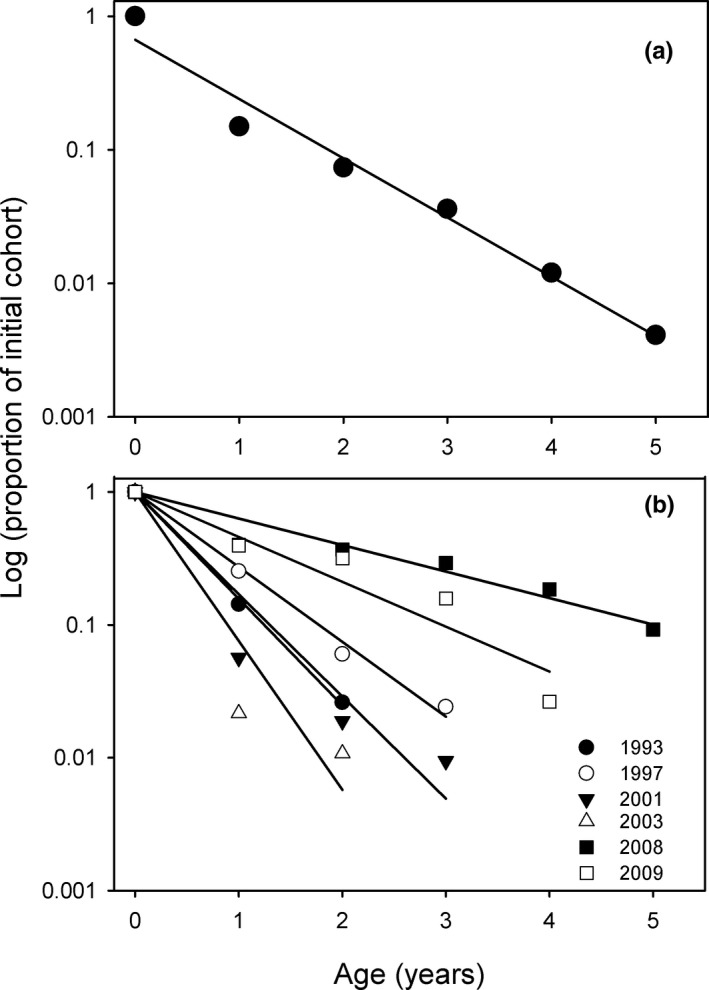
*Astragalus holmgreniorum* survivorship curve on a log‐linear scale: (a) with all cohorts pooled for the 15 cohorts with >10 individuals and at least 3 years of survivorship data: log (proportion surviving) = 0.4438(age)–1.75; adjusted *R* ^2^ = .971, df = 4, *p* = .0003, and (b) for six representative cohorts, showing the large differences among cohorts in the slope of the relationship between log (survivorship) and age, as demonstrated by the highly significant age by cohort interaction from analysis of covariance (*F*_14,19_ = 6.43, *p *< .0001)

## Data Availability

Field datasets that form the basis of the analyses in this manuscript are deposited in DRYAD at https://doi.org/10.5061/dryad.tdz08kq12.

## APPENDIX 1

### Vital rate calculations

In this appendix, we present in detail how the values for vital rates needed to parameterize the elements in the transition matrix were calculated. These methods are described as follows:

1. Plant survival and size class transitions
2. Reproductive output parameters
3. Seed bank transitions
4. Germination and nondormant seed carryover
5. Germinant survival to census

Vital rates were designated as invariant or variable. Measured or estimated values for each variable vital rate were calculated for each year of the field study and these values were averaged. This resulted in a table of means and variances for each variable vital rate needed to parameterize the elements in the model transition matrix. See Appendix 2, Table A2.1 for vital rate codes, means, and variances and Table A2.2 for the transition matrix with element definitions.

#### Plant survival and size class transitions

Plants were classified into three size classes based on field observations indicating that small plants (Size Class 1; <6 cm in diameter) very rarely flowered and did not set significant amounts of seed. Plants from 6 to 15 cm in diameter (Size Class 2) generally set seed only in favorable years, while plants >15 cm in diameter (Size Class 3) often set some seed even in poor years. This relationship between size class and seed production was confirmed using a dataset from Searle (2011). We did not consider further division of Size Class 2 or 3 because small sample sizes in many years would have made survival and size class transition probabilities less reliable.

Means and variances for plant survival and size class transitions were calculated directly from the 22‐year demography dataset. For each pair of years in the study, we first calculated survival, that is, the proportion of individuals in each of the three size classes in Year_(_ *_n_*_)_ that survived to Year_(_ *_n_*_+1)_. Of these surviving plants of each size class, we then calculated the proportion that remained in the same size class the following year (stasis) and the proportion that transitioned to each of the other two size classes.

One important feature of the beta distribution used to specify transitions in the matrix model from Morris and Doak (2002) that we implemented (see Appendix 2) is that it can only model binary alternatives. As our model included transitions among three size classes, it was necessary to decompose these probabilities into stepwise binary probabilities. For example, the transition from size class S1 to another size class is broken down into:

S1 to S1 (stasis)
S1 to notS1
S1 to S2NEW
S1 to S3NEW

To calculate the probability of the transition from S1 to S2 using these binary vital rates requires multiplying the probabilities (S1 to notS1) and (S1 to S2NEW) together. Thus for the matrix elements that quantify the transition from Size Class 1 to another size class each time step, the following survival and size class transition vital rates were combined:

**Table:**

Matrix element S1 to S1	(S1 survival) *(S1 to S1)
Matrix element S1 to S2	(S2 survival)*(S1 to notS1)*(S1 to S2New)
Matrix element S1 to S3	(S3 survival)*(S1 to notS1)*(S1 to S3New)

Matrix elements for the transitions from S2 to S3 and from S3 to S2 were calculated by combining survival and size class transitions in the manner described above. Matrix elements for transitions from S2 and S3 to S1 also included vital rates parallel to those discussed here, but because these transitions also included probabilities for recruitment of S1 plants from seeds produced in the current year by S2 and S3 plants, these matrix elements included additional terms as described later.

### Reproductive output parameters

Estimates of seed yield per plant for potentially reproductive individuals (size classes S2 and S3) each year were obtained by combining yearly flower count data from the demography study with estimates of seed production per flower from an independent dataset (Searle, 2011). Each year, every tagged S2 and S3 plant on the study site was scored as either flowering or nonflowering, and the number of flowers on each flowering plant was recorded. Mean flower counts per plant for S2 and S3 plants were then calculated for each year. To obtain an estimate of seed production per flower for each reproductive size class each year required an estimate of fruit set (fruits per flower) and seed production per fruit for each reproductive size class each year:

fruits/flower∗seeds/fruit=seeds/flower.

As part of a larger study, Searle (2011) quantified fruit set and seeds per fruit in the State Line population, which included the study site, in 2 years: 2009, a year with low growing season precipitation (February–May total 48 mm) and 2010, a year with above‐average growing season precipitation (February–May total 106 mm). We used regression based on these 2 years of data to develop a putative linear relationship between seeds per flower and growing season precipitation for S2 and S3 plants (Figure A1.1).

The resulting regression equations were used to estimate seeds per flower for flowering S2 and S3 flowering plant for each year of the study:

These equations were used along with yearly spring precipitation data to obtain estimates for mean seed number per flower each year of the study for each reproductive size class:

S2Seeds per Flower=0.06053∗(FMAM precipitation)‐2.1146

S3Seeds per Flower=0.03846∗(FMAM precipitation)+2.4557

Mean seeds per flower for S2 and S3 plants each year was then multiplied by mean flowers per plant to estimate yearly seed production:

Mean flowers/plant from field counts∗mean seeds/flower from regression=mean seeds per plant

This approach to estimating seeds per flower represented an effort to improve the precision of seed production estimates as compared to using a constant value for seeds per flower for both size classes and for every year of the study. We only had seeds per flower data from 2 years, so that the analysis has no associated error estimate and assumes a linear relationship. However, the fact that the 2 years of the reproductive output study were so different in terms of both spring precipitation and seed production per flower lends credence to this approach. Seed production per flower was a relatively small contributor to seed production calculations, which were much more heavily influenced by the yearly flower count data.

To obtain the long‐term mean and variance for seed production per plant of each reproductive size class for use in the model, these yearly means were then averaged. These reproductive output values for S2 and S3 plants are represented by the vital rates vrs(13) and vrs(14) in Table A2.1 and A2.2.

#### Seed bank transitions

Estimates for seed bank transitions were derived from a 6‐year seed retrieval experiment (Searle, 2011; A. Searle, unpublished data). The study was initiated in summer 2009 with freshly collected seeds from three populations. Thirty groups of 20 seeds from each seed population were packaged into 4‐cm^2^ nylon mesh bags. The bags were buried ca. 2 cm deep at each of the three sites. At each site two replicates, each consisting of one bag from each of the three seed populations, were included for each potential retrieval date for a total of five possible retrieval dates (for each seed population: 3 sites × 2 replicates × 5 retrieval dates = 30 bags).

Seeds were retrieved in early summer in three subsequent years: 2010 (year 1), 2012 (year 3), and 2015 (year 6). Two replicates were retrieved from each site in each of the first 2 years (6 bags per seed population per year), while all remaining bags were retrieved in 2015. Seeds that remained in each bag were quantified and tested for viability. Seeds that were not detectable or were present as seed coats only were assumed to have germinated. The remaining seeds were scarified with sandpaper, then imbibed in petri dishes at 30°C for 1 week. Seeds that germinated were quantified and removed, and the remaining ungerminated seeds were tested for viability using tetrazolium staining. Seeds that did not stain were scored as nonviable. Seeds lots used in the retrieval were also subjected to tetrazolium evaluation at the initiation of the experiment to provide a viability estimate. Initial viability averaged 95% in these late‐collected seed lots, but viability in the reproductive output studies described earlier was near 100%. All viable seeds in the seed bags were assumed to be dormant, based on the fact that adequate precipitation for full germination of nondormant seeds was received during every year of the retrieval study. This means that only dormant seeds would remain as viable seeds.

The retrieval data were first analyzed as a full factorial using analysis of covariance for binomial data in the SAS program GLIMMIX with seed population and site as the class variables and number of years post‐burial as the continuous variable. Because neither seed population nor site was significant, data were pooled by retrieval date for analysis using linear regression with number of years post‐burial as the continuous independent variable. The analyzed response variables were: (1) fraction of seeds remaining viable, that is, germinable after scarification plus viable using tetrazolium staining and (2) fraction of nonviable seeds. Initially viable seeds not in these two categories were assumed to have germinated; these were still present as empty seed coats in most cases. Intact nonviable seeds were also still present in the bags, likely because the bags spent very little time in a wet condition over the course of the study.

This analysis showed that the fraction of dormant seeds remaining in a cohort decreased linearly through time, that is, each year a constant fraction (11.1%) of the original seed cohort either lost dormancy or became nonviable (Figure A1.2). This line crossed the X‐axis at 9 years, the estimated maximum dormant seed bank longevity. Dormancy loss rate was insensitive to environmental variation. The absolute viability loss rate was also linear and insensitive to environmental variation and was estimated at 3% of the original cohort per year (Figure A1.2). This indicated that 8.1% of the original cohort became germinable each year.

In order to parameterize seed bank transitions in the model, it was necessary to know what fraction of the *remaining seeds* in a cohort would be maintained as dormant seeds at each seed age, what fraction would become nondormant, and what fraction would lose viability.

Table A1.1 shows how these fractions are calculated. Each year the fraction of the original cohort to remain dormant decreases by 11.1%; 8.1% of the original cohort becomes nondormant and 3.0% becomes nonviable. The first step is to calculate the fraction of the original cohort that persists as dormant seeds each year (ABSDFRAC_current year_) by subtracting 0.111 from the absolute number that remained the previous year (ABSDFRAC_previous year_). The fraction of the remaining seeds that persists as dormant seeds from one seed age to the next is:

$${\text{ABSDFRAC}}_{\text{current year}}/{\text{ABSDFRAC}}_{\text{previous year}}$$

This value, which is the transition probability that dormant seeds of a given age will remain in the dormant seed bank the following year, increases with seed age because each year there are fewer dormant seeds left, but a constant number is released from dormancy. For this reason, it is necessary to model dormant seed transitions as age‐specific. Similarly, the fraction of the original cohort to become nondormant each year (ABSNDFRAC) is also constant through time at 8.1%, but its model transition probability is:

$${\text{ABSNDFRAC}}_{\text{current year}}/{\text{ABSDFRAC}}_{\text{previous year}}$$

**TABLE A1.1:** Calculating dormant seed transitions to year‐older dormant seeds (DORM0‐DORM8) and dormant seed transitions to nondormant seeds (NDORM0 to NDORM9) for seeds of ages from 0 to 9 years

Seed age	ABS DFRAC	ABS NDFRAC	Abs TV‐Frac	Abs Tot Curr	REL DFRAC (DORM0‐DORM8)	REL NDFRAC (NDORM0‐NDORM9)	Rel TV‐ frac	Rel Tot Curr
0	0.889	0.081	0.03	1	0.889	0.081	0.030	1.000
1	0.778	0.081	0.03	0.889	0.875	0.091	0.034	1.000
2	0.667	0.081	0.03	0.778	0.857	0.104	0.039	1.000
3	0.556	0.081	0.03	0.667	0.834	0.121	0.045	1.000
4	0.445	0.081	0.03	0.556	0.800	0.146	0.054	1.000
5	0.334	0.081	0.03	0.445	0.751	0.182	0.067	1.000
6	0.223	0.081	0.03	0.334	0.668	0.243	0.090	1.000
7	0.112	0.081	0.03	0.223	0.502	0.363	0.135	1.000
8	0.001	0.081	0.03	0.112	0.000	0.723	0.268	0.991
9	0	0.001	0	0.001		1.000		1.000

Similarly, the fraction of the original cohort to become nondormant each year is also constant through time at 8.1%, but its transition probability for the model is 0.081/ABSDFRAC_previous year_, a number which increases with seed age, as the fraction of the original cohort is a larger fraction of the remaining seeds. Using these relative fractions as the transition probabilities for dormant to dormant and dormant to nondormant seed bank transitions takes viability loss in the seed bank into account, as the viability loss fraction is excluded from the calculations for each age.

Calculation of transition probabilities for shorter periods of seed bank persistence follows the identical procedure, except that the absolute fraction of seeds to become nondormant each year is increased. For example, for 5‐year persistence, the regression line in Figure A1.2 would cross the *Y*‐axis at 5 years, and 0.200 of the original seed cohort would become nondormant each year. Of these, 0.03 would lose viability and 0.17 would become nondormant. Substituting these values into the spreadsheet yields transition probabilities for DORM0‐DORM8 and corresponding values for NDORM0 though NDORM9 for use in the model, but values for DORM 5‐DORM8 and NDORM6‐NDORM9 would already be at zero.

#### Germination and nondormant seed carry‐over

Field retrieval experiments have demonstrated that most nondormant seeds germinate, at least in shallowly buried retrieval bags, except in years of very low winter‐spring precipitation (<50 mm). To include the possibility that nondormant seeds could carry over to the following year, we included the stage Nondormant in the model. Lacking detailed information on germination fraction as a function of precipitation, we adopted a simplified version. We set germination at 1.00 for years with >50 mm of winter‐spring precipitation and at 0 for years with <50 mm. Using these coarse estimates along with yearly precipitation data resulted in a mean germination fraction of 0.90 and a germination fraction variance of 0.085. This gives a reasonably realistic estimate of what might happen in the field, namely, most years have full or near‐full germination, but a few have very low or no germination. Each yearly time step, the model uses this probability distribution to calculate the fraction of nondormant seeds (NDORM0 through NDORM9) that will germinate (GFRAC) and the fraction that will carry over into the Nondormant stage (1‐GFRAC).

#### Germinant survival to census

Calculation of germinant survival to census requires knowledge of the number of seeds that germinate as well the number of seedlings that successfully recruit and are present as S1 plants at census in April. This is not the same as the number of seedlings that emerge, as many seeds may germinate but not produce emerged seedlings. This fraction (germinated seeds/recruited seedlings) cannot be obtained from observational data without accurate knowledge of numbers of nondormant seeds in the soil seed bank. We used an experimental dataset to measure germinant survival in an exceptionally favorable year. We carried out introduction seedings in fall 2018 at 10 sites in suitable unoccupied habitat (Meyer & Rominger, 2021). We used both unscarified seeds (expected to persist for 9 years and create yearly recruitment episodes) and scarified seeds (expected to germinate the year of the study with adequate precipitation) in separate treatments. The winter‐spring precipitation in 2018–2019 was far above average at 180 mm, and scarified seeds in retrieval bags at all ten sites germinated to 100%. Germinant survival in the scarified plots at each site could then be calculated directly:

germinant survival=number of recruits/number of scarified seeds planted

Averaged over the 10 sites, germinant survival averaged 0.24, that is, 24% of the planted scarified seeds were present as recruited seedlings in April, a very high level of recruitment success. To translate this single number into estimates of recruitment success each year of the demography study, we decided to use 0.24 as the maximum recruitment success possible, that is, with no further increase in recruitment success at precipitation values >180 mm. To anchor the lower end of the recruitment success curve, we set success at 0 when precipitation ≤50 mm, based on the demographic study showing no recruitment in such dry years (Figure A1.3).

To estimate germinant survival to the census for each year of the demographic study with precipitation ≤180 mm, we used the following equation based on Figure A1.3 above:

Germinant survival to census=0.0185∗(winter‐spring precipitation)‐0.092

Germinant survival for years with precipitation >180 mm was set at a constant 0.24. We then averaged these calculated values over the years of the demographic study. We obtained a mean value of 0.1007 and a variance of 0.085 for germinant survival to census to use as the probability distribution for this vital rate in the model.

## APPENDIX 2

### Population matrix modeling explanation

We used guidelines and MATLAB code provided in the book Quantitative Conservation Biology: Theory and Practice of Population Viability Analysis (Morris & Doak, 2002) to construct the population matrix model for *Astragalus holmgreniorum* that is presented in the main manuscript. Chapters 6, 7, and 8 of this book deal with building and executing populationmatrix models based on vital rates. The book was designed to be used by professionals not highly trained in matrix modeling and provides clear and understandable instructions for adapting the MATLAB code they include for use in population matrix modeling with any species of interest. Electronic files of the MATLAB code in the book were provided online ready for adaptation and use.

We used advice in Chapter 6 of Morris and Doak to examine our 22‐year demographic dataset and develop a life‐history diagram and its corresponding projection matrix, defining classes based on size for actively growing plants but using an age‐based classification for dormant seeds in the seed bank. We used the demographic dataset to calculate vital rates for each class each year as described in Appendix 1. For actively growing plants, we calculated survival, transition from one size class to another, and reproductive output. For current‐year seed production, we calculated the fraction that would germinate the same year, that would become nondormant but fail to germinate and be present as nondormant seeds the following year, and that would remain dormant and become part of the 1‐year‐old dormant seed bank the following year. Age‐specific transitions for seeds in the persistent seed bank as well as transitions to the nondormant seed fraction were also included. Transitions involving actively growing plants varied each year in response to environmental variation, while transitions in the dormant seed bank were treated as invariant, that is, not sensitive to environmental variation.

We calculated means and variances for the vital rates that were variable, and estimated or calculated values for invariant vital rates as described in Appendix 1. We then chose probability distributions for the vital rates to be used in the model. The table of vital rate means, variances, and statistical distributions that follows this section provide these values (Table A2.1).

As per Morris and Doak p. 275–282, we used the beta distribution rather than the normal distribution to model survival and transition probabilities, and the stretched beta distribution rather than the log‐normal distribution to model seed production. These distributions have the advantage of being bounded between 0 and 1 for the beta distribution and bounded between user‐defined minimum and maximum values for the stretched beta distribution. These limits prevent the model from choosing biologically unrealistic or even impossible values from the probability distribution for each time step. The mathematical calculations for the beta distribution can be cumbersome, but the book provides useful computational shortcuts and also MATLAB code for making these computations that can be embedded in the model program (betaval, Box 8.3; stretchbetaval, Box 8.5).

Another key component of a realistic population matrix model is the inclusion of covariances among vital rates. To accomplish this, Morris and Doak (p.282–305) provided a method for using within‐year and across‐year correlation matrices for variable vital rates to construct corrected matrices that can be used to pick random values with the correct degree of correlation from the beta or stretch beta distribution for each variable and that will include consideration of the degree of within‐year correlation, autocorrelation, and cross‐correlation among variables.

The first step for constructing the derived matrices is to check whether each original correlation matrix meets the matrix algebra assumptions that make its use possible in the model. Missing data, small numbers of observations and other irregularities can cause the matrix to contain unacceptable internal logical inconsistencies that must be corrected before the matrix is usable. Box 8.8 in Morris and Doak provides the MATLAB code to do this. These corrected matrices are then used in combination with the vital rate means, variances, and statistical distributions in a rather complex set of calculations to generate sets of within year, auto‐, and cross‐correlated vital rates (Box 8.9). The correlated vital rates calculation subroutine is then embedded into the program file that simulates population growth and extinction risk (Box 8.10).

In order to run the program in Box 8.10 for *Astragalus holmgreniorum*, we listed means and variances for vital rates in our model, specified statistical distributions for each vital rate, and referenced the transition matrix m file (Table A2.2) that defines each matrix element in terms of vital rates. We also loaded the within‐year and across‐year corrected correlation matrices. We then specified the vector of initial stage values, the quasi‐extinction threshold, the number of years to simulate, the number of correlated vital rates in the correlation matrix, the number of uncorrelated vital rates, the dimensions of the population transition matrix, and the number of iterations to perform.

The sections of the program calculate the mean population matrix and deterministic lambda, then create beta distributions that the program can use to select beta‐distributed random variables. This is followed by the complex set of calculations used to create correlated beta or stretch beta random variables. Finally, the program proceeds to the simulation of stochastic population growth and the calculation of extinction risk cumulative distribution functions. The program ends by specifying desired outputs of the model run.

**TABLE A2.1:** Vital rate codes and their names, means, variances, statistical distributions, and descriptions

Vital rate	Name	Mean	Variance	Distribution type	Description
vrs(1)	S1 TO S1 STASIS	0.310	0.117	Beta	Size Class 1 Stasis
vrs(2)	S1 NOT S1	0.690	0.117	Beta	Size Class 1 Not Stasis
vrs(3)	S1 S2 NEW	0.676	0.117	Beta	Size Class 1 to Size Class 2
vrs(4)	S1 S3 NEW	0.219	0.066	Beta	Size Class 1 to Size Class 3
vrs(5)	S2 TO S2 STASIS	0.308	0.096	Beta	Size Class 2 Stasis
vrs(6)	S2 NOT S2	0.692	0.096	Beta	Size Class 2 Not Stasis
vrs(7)	S2 S1 NEW	0.134	0.115	Beta	Size Class 2 to Size Class 1
vrs(8)	S2 S3 NEW	0.741	0.190	Beta	Size Class 2 to Size Class 3
vrs(9)	S3 TO S3 STASIS	0.728	0.137	Beta	Size Class 3 Stasis
vrs(10)	S3 NOT S3	0.235	0.127	Beta	Size Class 3 Not Stasis
vrs(11)	S3 TO S1 NEW	0.069	0.032	Beta	Size Class 3 to Size Class 1
vrs(12)	S3 TO S2 NEW	0.397	0.219	Beta	Size Class 3 to Size Class 2
vrs(13)	S2RO	24.8	2319	Stretch beta	Size Class 2 Reproductive Output
vrs(14)	S3RO	143.8	40,294	Stretch beta	Size Class3 Reproductive Output
vrs(15)	S1SURV	0.175	0.023	Beta	Size Class 1 Survival
vrs(16)	S2SURV	0.398	0.111	Beta	Size Class 2 Survival
vrs(17)	S3SURV	0.331	0.122	Beta	Size Class 3 Survival
vrs(18)	GFRAC	0.905	0.085	Beta	Germination Fraction
vrs(19)	GSURV	0.1007	0.085	Beta	Germinant Survival to Census
vrs(20)	DFRAC0	0.889	0	Invariant	Dormant Fraction Year 0
vrs(21)	DFRAC1	0.875	0	Invariant	Dormant Fraction Year 1
vrs(22)	DFRAC2	0.857	0	Invariant	Dormant Fraction Year 2
vrs(23)	DFRAC3	0.834	0	Invariant	Dormant Fraction Year 3
vrs(24)	DFRAC4	0.800	0	Invariant	Dormant Fraction Year 4
vrs(25)	DFRAC5	0.751	0	Invariant	Dormant Fraction Year 5
vrs(26)	DFRAC6	0.668	0	Invariant	Dormant Fraction Year 6
vrs(27)	DFRAC7	0.502	0	Invariant	Dormant Fraction Year 7
vrs(28)	DFRAC8	0	0	Invariant	Dormant Fraction Year 8
vrs(29)	ND1FRAC0	0.081	0	Invariant	Nondormant Fraction Year 0
vrs(30)	ND1FRAC1	0.091	0	Invariant	Nondormant Fraction Year 1
vrs(31)	ND1FRAC2	0.104	0	Invariant	Nondormant Fraction Year 2
vrs(32)	ND1FRAC3	0.121	0	Invariant	Nondormant Fraction Year 3
vrs(33)	ND1FRAC4	0.146	0	Invariant	Nondormant Fraction Year 4
vrs(34)	ND1FRAC5	0.182	0	Invariant	Nondormant Fraction Year 5
vrs(35)	ND1FRAC6	0.242	0	Invariant	Nondormant Fraction Year 6
vrs(36)	ND1FRAC7	0.363	0	Invariant	Nondormant Fraction Year 7
vrs(37)	ND1FRAC8	0.723	0	Invariant	Nondormant Fraction Year 8
vrs(38)	ND1FRAC9	1.00	0	Invariant	Nondormant Fraction Year9
vrs(39)	SRS	0.80	0	Invariant	Seed rain survival

**TABLE A2.2:** Transition matrix defining the vital rate computations for each of the matrix elements (stage transitions) in the model

	SB1	S1	S2	S3	SB2	SB3	SB4
SB1	0	0	vrs(13)*vrs(39)*vrs(20)	vrs(14)*vrs(39)*vrs(20)	0	0	0
S1	vrs(30)*vrs(18)*vrs(19)	vrs(15)*vrs(1)	(vrs(16)*vrs(6)*vrs(7))+ (vrs(13)*vrs(39)*vrs(29)*vrs(18)*vrs(19))	(vrs(17)*vrs(10)*vrs(11))+ (vrs(14)*vrs(39)*vrs(29)*vrs(18)*vrs(19))	vrs(31)*vrs(18)*vrs(19)	vrs(32)*vrs(18)*vrs(19)	vrs(33)*vrs(18)*vrs(19)
S2	0	vrs(15)*vrs(2)*vrs(3)	vrs(16)*vrs(5)	vrs(17)*vrs(10)*vrs(12)	0	0	0
S3	0	vrs(15)*vrs(2)*vrs(4)	vrs(16)*vrs(6)*vrs(8)	vrs(17)*vrs(9)	0	0	
SB2	vrs(21)	0	0	0	0	0	0
SB3	0	0	0	0	vrs(22)	0	
SB4	0	0	0	0	0	vrs(23)	0
SB5	0	0	0	0	0	0	vrs(24)
SB6	0	0	0	0	0	0	0
SB7	0	0	0	0	0	0	0
SB8	0	0	0	0	0	0	0
SB9	0	0	0	0	0	0	0
ND	vrs(30)*(1‐vrs(18))	0	vrs(13)*vrs(39)*vrs(29)*(1‐vrs(18))	vrs(14)*vrs(39)*vrs(29)*(1‐vrs(18))	vrs(31)*(1‐vrs(18))	vrs(32)*(1‐vrs(18))	vrs(33)*(1‐vrs(18))

	SB5	SB6	SB7	SB8	SB9	ND
SB1	0	0	0	0	0	0
S1	vrs(34)*vrs(18)*vrs(19)	vrs(35)*vrs(18)*vrs(19)	vrs(36)*vrs(18)*vrs(19)	vrs(37)*vrs(18)*vrs(19)	vrs(38)*vrs(18)*vrs(19)	vrs(18)*vrs(19)
S2	0	0	0	0	0	0
S3	0	0	0	0	0	0
SB2	0	0	0	0	0	0
SB3	0	0	0	0	0	0
SB4	0	0	0	0	0	0
SB5	0	0	0	0	0	0
SB6	vrs(25)	0	0	0	0	0
SB7	0	vrs(26)	0	0	0	0
SB8	0	0	vrs(27)	0	0	1‐vrs(18)]
SB9	0	0	0	vrs(28)	0	
ND	vrs(34)*(1‐vrs(18))	vrs(35)*(1‐vrs(18))	vrs(36)*(1‐vrs(18))	vrs(37)*(1‐vrs(18))	vrs(38)*(1‐vrs(18))	

## APPENDIX 3

### Survivorship analysis

Our population matrix model for *Astragalus holmgreniorum* is a stage‐based model, but our understanding the life‐history strategy of this species hinges on the relationship between environmental quality and the relationship of adult survival with age. Mean survivorship was log‐linear, that is, mortality risk was constant on average, especially after the first year (Deevey, 1947; Figure A3.1a). This suggests that mortality is environmentally mediated and not a consequence of senescence in older plants, which would tend to result in increased mortality risk with age.

The log‐linear relationship of survivorship with age was also evident for individual cohorts, but the slope of the line relating log survivorship to time varied dramatically among the six cohorts with sufficient data (Figure A3.1b). The 2008 cohort still had survivors at 5 years, whereas the 2003 cohort had almost no survivors at 2 years. The difference in survivorship among cohorts probably depends primarily on differences in year quality in the sequence of years following recruitment, specifically on the adequacy of winter precipitation.

## References

[ece38301-bib-0001] Augspurger, C. K. , & Salk, C. F. (2017). Constraints of cold and shade on the phenology of spring ephemeral herb species. Journal of Ecology, 105, 246–254. 10.1111/1365-2745.12651

[ece38301-bib-0002] Barneby, R. C. (1980). Dragma Hippomanicum V: Two new astragali from intermountain United States. Brittonia, 32, 24–29. 10.2307/2806212

[ece38301-bib-0003] Boyce, M. S. , Haridas, C. V. , Lee, C. T. , & NCEAS Stochastic Demography Working Group (2006). Demography in an increasingly variable world. Trends in Ecology andEvolution, 32, 141–148. 10.1016/j.tree.2005.11.018 16701490

[ece38301-bib-0004] Caswell, H. (2001). Matrix population models: Construction analysis and interpretation, 2nd ed. Sinauer.

[ece38301-bib-0005] Crone, E. E. , Menges, E. S. , Ellis, M. M. , Bell, T. , Bierzychudek, P. , Ehrlén, J. , Kaye, T. N. , Knight, T. M. , Lesica, P. , Morris, W. F. , Oostermeijer, G. , Quintana‐Ascencio, P. F. , Stanley, A. , Ticktin, T. , Valverde, T. , & Williams, J. L. (2011). How do plant ecologists use matrix population models? Ecology Letters, 14, 1–8. 10.1111/j.1461-0248.2010.01540.x 21070554

[ece38301-bib-0006] Danin, A. , & Orshan, G. (1990). The distribution of Raunkiaer life forms in Israel in relation to the environment. Journal of Vegetation Science, 1, 41–48. 10.2307/3236051

[ece38301-bib-0007] Deevey, E. S., Jr. (1947). Life tables for natural populations of animals. The Quarterly Review of Biology, 22, 283–314. 10.1086/395888 18921802

[ece38301-bib-0008] Drake, J. M. (2005). Population effects of increased climate variation. Proceedings of the Royal Society B: Biological Sciences, 272, 1823–1827. 10.1098/rspb.2005.3148 PMC1559868 16096095

[ece38301-bib-0009] Fragman, O. , & Shmida, A. (1997). Diversity and adaptation of wild geophytes along an aridity gradient in Israel. Acta Horticulturae, 430, 795–802. 10.17660/ActaHortic.1997.430.127

[ece38301-bib-0010] Gadgil, M. , & Solbrig, O. T. (1972). The concept of r‐ and K‐selection: Evidence from wild flowers and some theoretical considerations. American Naturalist, 106, 14–31. 10.1086/282748

[ece38301-bib-0011] Geiger, R. , Aron, R. H. , & Todhunter, P. (2009). The climate near the ground. Rowman and Littlefield.

[ece38301-bib-0012] Gremer, J. R. , & Sala, A. (2013). It is risky out there: The costs of emergence and the benefits of prolonged dormancy. Oecologia, 172, 937–947. 10.1007/s00442-012-2557-8. 23274621

[ece38301-bib-0013] Grime, J. P. (1977). Evidence for the existence of three primary strategies in plants and its relevance to ecological and evolutionary theory. American Naturalist, 111, 1169–1194. 10.1086/283244

[ece38301-bib-0014] Houghton, S. , Stevens, M. T. , & Meyer, S. E. (2020). Pods as sails but not as boats: Dispersal ecology of a habitat‐restricted desert milkvetch. American Journal of Botany, 107, 864–875. 10.1002/ajb2.1473 32462674

[ece38301-bib-0015] Lapointe, L. (2001). How phenology influences physiology in deciduous forest spring ephemerals. Physiologia Plantarum, 113, 151–157. 12060291 10.1034/j.1399-3054.2001.1130201.x

[ece38301-bib-0016] Lawson, C. R. , Vindenes, Y. , Bailey, L. , & van de Pol, M. (2015). Environmental variation and population responses to global change. Ecology Letters, 18, 724–736. 10.1111/ele.12437 25900148

[ece38301-bib-0017] Lewontin, R. C. , & Cohen, D. (1969). On population growth in a randomly varying environment. Proceedings of the National Academy of Sciences of the United States of America, 62, 1056–1060. 10.1073/pnas.62.4.1056 5256406 PMC223613

[ece38301-bib-0018] Meyer, S. E. , & Rominger, K. R. (2021). Chapter 3. *Astragalus holmgreniorum* introduction seedings. In S. E. Meyer & M. Stevens (Eds.), Annual report to the USDI Bureau of Land Management Utah State Office for Interagency Agreement No. IA‐18‐PG00120 FY2020. On file at the USDI Bureau of Land Management Utah State Office, Salt Lake City, Utah.

[ece38301-bib-0019] Morris, W. F. , & Doak, D. F. (2002). Quantitative conservation biology: the theory and practice of population viability analysis. Sinauer Associates.

[ece38301-bib-0020] Morris, W. F. , & Doak, D. F. (2004). Buffering of life histories against environmental stochasticity: accounting for a spurious correlation between the variabilities of vital rates and their contributions to fitness. American Naturalist, 163, 579–590. 10.1086/382550 15122504

[ece38301-bib-0021] Pake, C. E. , & Venable, D. L. (1996). Seed banks in desert annuals: Implications for persistence and coexistence in variable environments. Ecology, 77, 1427–1435. 10.2307/2265540

[ece38301-bib-0022] Peguero‐Pina, J. J. , Vilagrosa, A. , Alonso‐Forn, D. , Ferrio, J. P. , Sancho‐Knapik, D. , & Gil‐Pelegrín, E. (2020). Living in drylands: Functional adaptations of trees and shrubs to cope with high temperatures and water scarcity. Forests, 11, 1028. 10.3390/f11101028

[ece38301-bib-0023] Pianka, E. R. (1970). On r‐and K‐selection. American Naturalist, 104, 592–597. 10.1086/282697

[ece38301-bib-0024] Pierce, S. , Negreiros, D. , Cerabolini, B. E. L. , Kattge, J. , Díaz, S. , Kleyer, M. , Shipley, B. , Wright, S. J. , Soudzilovskaia, N. A. , Onipchenko, V. G. , van Bodegom, P. M. , Frenette‐Dussault, C. , Weiher, E. , Pinho, B. X. , Cornelissen, J. H. C. , Grime, J. P. , Thompson, K. , Hunt, R. , Wilson, P. J. , & Tampucci, D. (2017). A global method for calculating plant CSR ecological strategies applied across biomes world‐wide. Functional Ecology, 31, 444–457. 10.1111/1365-2435.12722

[ece38301-bib-0025] Rabinowitz, D. (1981). Seven forms of rarity. In H. Synge (Ed.), The biological aspects of rare plant conservation (pp. 205–217). Wiley.

[ece38301-bib-0026] Rominger, K. R. , Meyer, S. E. , Van Buren, R. , & Searle, A. B. (2019). Phenological patterns in the desert spring ephemeral Astragalus holmgreniorum Barneby (Fabaceae). Western North American Naturalist, 79, 308–322. 10.3398/064.079.0303

[ece38301-bib-0027] Salguero‐Gómez, R. (2017). Applications of the fast–slow continuum and reproductive strategy framework of plant life histories. New Phytologist, 213, 1618–1624. 10.1111/nph.14289 27864957

[ece38301-bib-0028] Schultz, B. S. , Meyer, S. E. , DeNittis, A. , & Rominger, K. R. (2021). Growing an endangered desert milkvetch for container seed production. Native Plants Journal, 22, 162–175. 10.3368/npj.22.2.162

[ece38301-bib-0029] Searle, A. B. (2011). Reproductive success and soil seed bank characteristics of *Astragalus* *ampullarioides* and *A*. *holmgreniorum* (Fabaceae): two rare endemics of southwestern Utah. M.S. thesis. Brigham Young University, Provo, Utah.

[ece38301-bib-0030] Searle, A. B. , & Meyer, S. E. (2020). Cattle trampling increases dormant season mortality of a globally endangered desert milkvetch. Journal for Nature Conservation, 56, 125868. 10.1016/j.jnc.2020.125868

[ece38301-bib-0031] Tepedino, V. J. (2005). Final report: Reproduction and pollination of two rare species of *Astragalus* from Washington County, Southern Utah: *A*. *holmgreniorum* and *A*. *ampullarioides* . Report on file at the USDA_ARS Pollinating Insect Biology, Management, Systematics Research Laboratory, Logan, Utah.

[ece38301-bib-0032] Tuljapurkar, S. D. , & Orzack, S. H. (1980). Population dynamics in variable environments. I. Long‐run growth rates and extinction. Theoretical Population Biology, 18, 314–342. 10.1016/0040-5809(80)90057-X

[ece38301-bib-0033] US Fish and Wildlife Service (2001). Endangered and threatened wildlife and plants: Determination of endangered status for *Astragalus* *holmgreniorum* (Holmgren milk‐vetch) and *Astragalus* *ampullarioides* (Shivwits milk‐vetch). Federal Register, 66, 49560–49567.

[ece38301-bib-0034] US Fish and Wildlife Service (2006). *Astragalus* *holmgreniorum* (Holmgren milk‐vetch) and *Astragalus* *ampullarioides* (Shivwits milk‐vetch) recovery plan. U S Department of Interior, Fish and Wildlife Service.

[ece38301-bib-0035] Van Buren, R. , & Harper, K. T. (2003). Demographic and environmental relations of two rare *Astragalus* species endemic to Washington County, Utah: *Astragalus* *holmgreniorum* and A. *ampullarioides* . Western North American Naturalist, 63, 236–243.

[ece38301-bib-0036] Whittaker, R. H. , & Niering, W. A. (1964). Vegetation of the Santa Catalina Mountains, Arizona. I. Ecological classification and distribution of species. Journal of the Arizona Academy of Science, 3, 9–34.

